# Interaction of field realistic doses of clothianidin and *Varroa destructor* parasitism on adult honey bee *(Apis mellifera L*.*)* health and neural gene expression, and antagonistic effects on differentially expressed genes

**DOI:** 10.1371/journal.pone.0229030

**Published:** 2020-02-20

**Authors:** Nuria Morfin, Paul H. Goodwin, Ernesto Guzman-Novoa

**Affiliations:** School of Environmental Sciences, University of Guelph, Guelph, Ontario, Canada; University of California San Diego, UNITED STATES

## Abstract

While many studies have examined the effects of neonicotinoid insecticides and the parasitic mite *Varroa destructor* on honey bees (*Apis mellifera*), more information on the combined effects of such stressors on gene expression, including neural related genes, and their impact on biological pathways is needed. This study analyzed the effects of field realistic concentrations of the neonicotinoid clothianidin on adult bees infested and not infested with *V*. *destructor* over 21 consecutive days and then determined bee survivorship, weight, deformed wing virus (DWV) levels and gene expression. *V*. *destructor* parasitism with or without clothianidin exposure was significantly associated with decreased survivorship, weight loss and higher DWV levels, while clothianidin exposure was only associated with higher levels of DWV. Expression analysis of the neural genes *AmNlg-1*, *BlCh* and *AmAChE-2* showed that *V*. *destructor* caused a significant down-regulation of all of them, whereas clothianidin caused a significant down-regulation of only *AmNrx-1* and *BlCh*. An interaction was only detected for *AmNrx-1* expression. RNAseq analysis showed that clothianidin exposure resulted in 6.5 times more up-regulated differentially expressed genes (DEGs) than *V*. *destructor* alone and 123 times more than clothianidin combined with *V*. *destructor*. Similar results were obtained with down-regulated DEGs, except for a higher number of DEGs shared between *V*. *destructor* and the combined stressors. KEGG (Kyoto Encyclopedia of Genes and Genomes) biological pathway analysis of the DEGs showed that the stressor linked to the highest number of KEGG pathways was clothianidin, followed by *V*. *destructor*, and then considerably fewer number of KEGG pathways with the combined stressors. The reduced numbers of DEGs and KEGG pathways associated with the DEGs for the combined stressors compared to the stressors alone indicates that the interaction of the stressors is not additive or synergistic, but antagonistic. The possible implications of the antagonistic effect on the number of DEGs are discussed.

## Introduction

Honey bees (*Apis mellifera* L.), the most important pollinators of agricultural crops and wild plants [1,2], have been afflicted by high rates of colony mortality in recent years in North America [3,4]. Many researchers have proposed that the interaction of multiple stressors is a likely explanation of extreme colony losses. Two of the factors often associated with honey bee mortality are the parasitic mite *Varroa destructor* and exposure to pesticides, particularly neonicotinoid insecticides [5,6].

*V*. *destructor* parasitism is a very serious health problem for honey bees as the mite not only damages the bee by feeding on the hemolymph and fat tissue [7,8], but it also vectors a number of viruses with deformed wing virus (DWV) being the most pathogenic to honey bees [9,10]. Additionally, *V*. *destructor* impairs the humoral and cellular responses of the immune system of honey bees [11,12,13]. Furthermore, *V*. *destructor* has been reported to affect the neural processes of honey bees by impairing grooming behaviour [14], non-associative learning [15] and homing ability in worker bees [16]. A gene that has been associated with neurodegeneration in *Drosohila melanogaster*, *bluecheese (BlCh)*, has been found to be affected by *V*. *destructor* parasitism in honey bees [17,18], but there are no reports on the combined effects of *V*. *destructor* and abiotic stressors, such as neurotoxins, on the gene’s expression.

Neonicotinoid insecticides are the most widely used systemic pesticides globally [19]; these pesticides are neurotoxins that act as agonist of nicotinic acetylcholine receptors (nAChRs) of the central nervous system of insects [20]. Acute neonicotinoid poisoning results in high honey bee mortality [21], but it has been argued that the field realistic levels at which pollinators are exposed by consuming pollen or nectar of treated plants is not detrimental to their health [22]. Several studies have not found damaging effects when honey bees are exposed to sublethal doses of neonicotinoids [23, 24, 25, 26]. However, other studies have reported that sublethal doses of neonicotinoid insecticides negatively affect bee olfaction [27], learning and homing behaviour [28, 29], foraging behaviour [29,30], and queen fecundity [31]. Also, sublethal neonicotinoid exposure can be detrimental to hygienic and grooming behaviours [14, 29, 32], longevity [32], humoral immunity [33] cellular immunity [34], hypopharyngeal gland development [35] and Malpighian tubule system function [36]. Sublethal doses of neonicotinoids can affect gene transcription, such as the down-regulation of differentially expressed genes (DEGs) for biosynthetic processes detected by RNAseq [37] and up-regulation of acetylcholinesterase (*AChE*) detected by qRT-PCR [38, 39]. Acetylcholine has been associated with learning in honey bees [40], and thus the effect of agonist of nAChR, such as clothianidin, with its subsequent effect on the enzyme acetylcholinesterase could have detrimental effects on bee behaviour. The activity of acetylcholinesterase has been proposed to be useful marker for the effect of nAChR agonists in bees and other insects [41]. Also, two other genes that code for pre and post synaptic proteins, neurexin and neuroligin, have been linked to associative learning in bees [42]. However, the effects of neonicotinoid insecticides on their expression in honey bees are unknown.

Thus far, studies about the impact of the combined effects of *V*. *destructor* and sublethal doses of neonicotinoids on honey bees, include research on *V*. *destructor* and imidacloprid showing reduced flying distance and increased body mass of bees [43], *V*. *destructor* and clothianidin demonstrating a decreased proportion of bees performing grooming behaviour [14], and *V*. *destructor* with clothianidin or thiamethoxam revealing increased overwintering colony mortality [44]. In addition, a few other studies have focused on the interaction between the sublethal exposure to neonicotinoids and other pathogens. For example, the combination of the fungus *Nosema ceranae* and imidacloprid resulted in increased mortality in bees [45], the combination of the bacterium *Paenibacillus larvae* and thiamethoxam resulted in increased mortality and decreased learning and memory [46], and the combination of black queen cell virus (BQCV) and clothianidin decreased larval survival [47]. These results indicate that there are detrimental effects on the health of honey bees when parasites and pathogens are combined with sublethal exposures to neonicotinoid insecticides, but the interaction can have different outcomes, depending on the biological agent under study, the dose and type of neonicotinoid insecticide, and the aspects of honey bee health measured. Thus, additional studies on the effects of pathogens and neonicotinoids at field realistic doses are needed to better understand how abiotic and biotic stressors interact and affect honey bee health, including assessing their effects on gene expression and biological pathways.

As *V*. *destructor* and neonicotinoids are two stressors of honey bees that have been proposed to be associated with extreme colony losses, this study was conducted to assess the interaction of field realistic doses of clothianidin, one of the most widely used neonicotinoids, on adult bees with *V*. *destructor* parasitism.

Adult bees were exposed to each stressor alone or both stressors for 21 days and then evaluated for survivorship, weight, DWV levels, expression of the neural related genes *neurexin (AmNrx-1*), *neuroligin (AmNlg-1)*, *bluecheese (BlCh)* and *acetylcholinesterase (AmAChE-2)*, as well as genome level gene expression using RNA sequencing (RNAseq) with an examination of the associated biological pathways.

## Materials and methods

### Ethics statement

No permits were required to conduct the research. The research and the analyses were conducted under the supervision of researchers of the Honey Bee Research Centre, University of Guelph in Guelph, ON, Canada. Beekeeping practices were done in accordance with the Ontario Ministry of Agriculture, Food and Rural Affairs (OMAFRA) bio-safety regulations.

### Source of honey bees and *V*. *destructor*

Honey bees were obtained from colonies of the Buckfast strain kept at the Honey Bee Research Centre, University of Guelph, ON, Canada (N43°32'12.883", W80°12'50.875"). The queens that provided the newly emerged bees were mated under controlled conditions in isolation at Thorah Island, Simcoe, ON, to guarantee the purity and uniformity of the Buckfast strain. The colonies were not treated or exposed to pesticides before or during the study.

Female varroa mites were collected from highly infested colonies, kept at the Honey Bee Research Centre (N43°32'12.883", W80°12'50.875"), as per Arechavaleta and Guzman-Novoa [48]. The collected mites were transferred to a Petri dish (Fisher Scientific sterile 100 mm X 15 mm polystyrene Petri dish) using a fine paint brush. The bottom of the Petri dish contained a Whatman No. 1 filter paper with a moistened cotton ball (0.25 cm diameter) to provide a humid environment. The mites were used within one hour after collection to infest honey bees for the study.

### Clothianidin doses

Field realistic concentrations of clothianidin were calculated based on 25.5–39 mg of nectar typically consumed by a honey bee per day [49], and the concentration of clothianidin in canola nectar, which is between 0.001–0.0086 ng/mg [50, 51]. Thus, the amount of clothianidin that a honey bee could consume in the nectar in a day may range between 0.03 and 0.34 ng ($\bar{x}$ = 0.15±0.06), which is 10 to 117 times lower than the reported oral LD_50_ for bees in 24 h (3.53 ng of clothianidin per bee) [52]. Thus, four concentrations of clothianidin (Sigma Aldrich®, Oakville, ON, Canada), 0, 9x10^-4^, 4.2x10-^3^, and 1x10^-2^ ng clothianidin/μl were used in this experiment considering a daily consumption of sugar syrup of 30–33 μl per bee per day [53, 54].

### Exposure to clothianidin and/or *V*. *destructor* and determination of bee survivorship and weight

To obtain newly emerged bees (<24 h), frames with emerging brood from source colonies were maintained overnight inside screened emerging cages (50.3 X 7.3 X 25.2 cm) in an incubator (35° C, 60% RH). The next morning, groups of 40 randomly collected bees were assigned to a treatment and placed in sterilized hoarding cages (12.7 X 8.5 X 14.5 cm) providing them H_2_O and 50% sucrose syrup in 20 ml gravity feeders. One cage per treatment was used and the cages were maintained in an incubator (35° C, 60% RH) for 21 consecutive days. The treatments consisted of a control (0 ng/μl), clothianidin only (9x10^-4^ ng/μl, 4.2x10^-3^ ng/μl or 1x10^-2^ ng/μl), parasite only (0 ng/μl + *V*. *destructor*), or both stressors (9x10^-4^ ng/ μl + *V*. *destructor*, 4.2x10^-3^ ng/μl + *V*. *destructor* and 1x10^-2^ ng/μl + *V*. *destructor*) [14]. The doses of clothianidin were delivered in sucrose syrup, which was available to the bees *ad libitum*, and its consumption was monitored every four days to confirm that the bees received the intended treatments. The feeders were weighed and refilled every four days to calculate the syrup consumed per bee per day. The consumption of sugar syrup by the bees was within the ranges reported in the literature (28.4±0.70 μl) [53, 54]. For the treatments with *V*. *destructor*, the newly emerged bees were held by their wings, and one *V*. *destructor* female was taken from a Petri dish using a fine paintbrush, placed on the abdomen or thorax of a bee and observed until it was visually verified that the mite was attached to the bee’s body. Then the bee was placed in its hoarding cage. To determine survivorship and mortality, dead bees were counted and removed from each cage daily until day 21. On day 21, live bees from each group were counted and then weighed with an analytical balance (Denver Instrument®, Bohemia, NY, USA). Three replicates of the experiment were conducted.

### RNA extraction and cDNA synthesis

Total RNA was extracted from five bees per sample for each of the repetitions as per Chen et al. [55]. The quality and concentration of the RNA were measured by determining the absorbance ratio (260/280 nm and 260/230 nm) using a spectrophotometer (NanodropLite^™^, Thermo Scientific, Mississauga, ON, Canada). Values between 1.8–2.0 for 260/280 nm and values between 2.0–2.2 for 260/230 nm were considered acceptable for purity, indicating no significant presence of contaminants, such as phenol or proteins. cDNA was prepared using a RevertAid^™^ H Minus First Strand cDNA Synthesis Kit (Fermentas, Burlington ON, CA) following the manufacturer’s instructions using 2,000 ng of RNA for each sample.

### DWV quantification

Reactions consisted of 2 μl cDNA, 0.4 μl each DWV primer (200 nM), 10 μl PowerUp^™^ Sybrgreen (2X) (Applied Biosystems, Foster City, CA, USA) and 7.2 μl nuclease free H_2_O. DWV primers reported by Di Prisco et al. [56] were used (Table A in S1 File). As a negative control, nuclease free H_2_O was included instead of cDNA, and a positive control from previously identified DWV positive bee samples by qRT-PCR were included. PCR conditions consisted of one cycle at 48° C for 15 min, one at 95° C for 10 min, 40 cycles at 95° C for 15 s and 60° C for 60 s, followed by one cycle at 68° C for 7 min.

Calibration curves to convert Ct values to DWV genome copies were done using 300 bp gBlocks® (Integrated DNA Technologies, Coralville, IA, USA) that included the sequence of the forward primer, amplicon and reverse primer. gBlocks® have been used for the construction of calibration curves for the quantification of target sequences in different models, such as honey bees and fresh water fish [14, 57, 58]. The lyophilized gBlocks® was diluted with 20 μl of ds H_2_O to obtain an initial concentration of 10 ng/μl that was used to make serial dilutions from 10^9^ to 10^1^ copies. Using a plot of Ct values versus DWV copy number (log_10_), a linear equation was used to calculate the DWV genome copy numbers per μg of RNA for each of the samples of interest [59].

### Gene expression analysis from quantitative real time-PCR

The neural genes of interest (*AmNrx-1*, *AmNlg*, *BlCh* and *AChE-2*) were amplified using primers from previous studies or designed for this study (Table A in S1 File). The primers used to amplify *AmAChE-2* (GenBank, accession No. KU532289) were designed with NCBI Primer-BLAST. Of the candidate constitutive reference genes, *β-actin*, *AmRPS5* and *AmGAPD2*, *AmRPS5* was selected as the reference gene from the stability values as determined by NormFinder [60]. The FASTA sequences of the selected genes were obtained from GeneBank® to design synthetic gene fragments (gBlocks®; Integrated DNA Technologies, Coralville, IA, USA) of 300 bp. The gBlocks® containing the sequences of the forward primer, amplicon sequence and reverse primer, were used to make 10-fold serial dilutions (10^9^−10^1^ copies) to generate standard curves used to optimize the qRT-PCR conditions. To confirm the specificity of the target gene, a melt curve analysis was included after each qRT-PCR run.

qRT-PCR was performed with a BioRad CFX96^™^ thermocycler (Bio-Rad Laboratories, Mississauga, ON, CA) with PowerUp^™^ Sybrgreen (2X) (Applied Biosystems, Foster City, CA, USA). The qRT-PCR reactions were carried out in well plates (Diamed®, Mississauga, ON, CA). Reactions consisted of 2 μl of cDNA, 0.6–1.4 μl primers (300 to 700 nM depending on the target optimization protocol), 10 μl of PowerUp^™^ Sybrgreen (2X), and 5.2–6.8 μl of nuclease free H_2_O. A three-step cycling protocol was used for *BlCh*, and consisted of a UDG (Uracil-DNA glycosylase) pre-treatment step at 50° C for 2 min, denaturation at 95° C for 10 min, and then 40 cycles of 95° C for 15 s, 60° C for 60 s and 72° C for 30 s. A two-step cycling protocol was used for *AmNrx-1*, and *AmNlg-*,*1* and *AmAChE-2*, and consisted of an UDG (Uracil-DNA glycosylase) pre-treatment at 50° C for 2 min, denaturation for 95° C for 10 min, and then 40 cycles of 95° C for 15 s and 60° C for 60 s. A negative control, nuclease free H_2_O was used instead of cDNA, and a positive control using the corresponding gBlock® dilution was used in each qRT-PCR run. The expression level of the target gene was normalized to the expression level of the reference gene using the 2^-ΔΔ^ (Livak) method [61] with the non-treated control group as calibrator. The Bio-Rad CFX Manager® software (Bio-Rad Laboratories, Mississauga, ON, CA) was used to calculate the expression ratio.

### RNA sequencing

Total RNA was extracted from 24 bees from each biological repetition, using the TRIzol® reagent (Fisher Scientific, Mississauga, ON, CA) following the manufacturer’s instructions. The RNA samples were sent to McGill University (Génome Québec Innovation Centre, Montreal, QC, CA) to perform a high throughput sequencing analysis using a HiSeq2500 v4 (Illumina, San Diego, CA, USA). For quality check, the OD 260/280 ratio was determined to be between 1.8 and 2.0. Library preparation for Illumina sequencing was done using the NEB kit Illumina (San Diego, CA, USA) and the KAPA kit (Roche, Mississauga, ON, CA), according to the manufacturer’s instructions. Sequencing was performed as 125 bp, paired-end reads.

Bioinformatic analysis was done at the Canadian Centre for Computational Genomics (C3G). The Illumina CASAVA pipeline was used for base calling. Trimming and clipping of adapters were done with Trimmomatic software [62]. Read sets were then aligned to a reference genome of the honey bee, *Apis mellifera* (ftp://ftp.ncbi.nlm.nih.gov/genomes/Apis_mellifera) (ver Amel_4.5) using STAR [63]. The RNA-Seq fragment counts were normalized based on their length. Aligned RNAseq reads were assembled into transcripts, and their abundance in fragments per kilobase of exon per million fragments mapped (FPKM) was determined with Cufflinks [64], which was also used to detect unknown or novel transcripts or isoforms. Differential gene analysis (DGA) was done using DESeq R Bioconductor package [65], and edgeR Bioconductor package [66] based on the raw read counts generated by HTSeq (http://htseq.readthedocs.io/en/release_0.9.0/). Transcript expression levels and test for significant differences (P<0.05) was calculated with Cuffdiff [64].

Biological pathways of the DEGs was determined by the KASS-KEGG automatic annotation server [67] with the Kyoto Encyclopaedia of Genes and Genomes (KEGG) [68]. Venn diagrams were created using the Bioinformatics and Evolutionary Genomics website (http://bioinformatics.psb.ugent.be/cgi-bin/liste/Venn/calculate_venn.htpl).

### Statistical analyses

The data from surviving bees were subjected to survival analysis using the Kaplan-Meier log rank test, and all curves were compared using pairwise comparisons, with an adjusted p value and log rank (Mantel Cox) tests. Data for the weight of 21-day old bees were assessed for normality using the Shapiro Wilk test. Weight data were log_10_ transformed before subjecting them to a two-way ANOVA and Tukey HSD tests (α of 0.05). Fold changes of relative gene expression were log_2_ transformed before subjecting them to a two-way ANOVA and Tukey HSD tests because they did not comply with normality based on the Shapiro Wilk test. DWV data were subjected to a Kruskal Wallis and Conover-Iman procedure, since they were not normally distributed and could not be transformed. The relative gene expression of *AmNrx-1*, *AmNlg-1*, *BlCh*, and *AmAChE-2* was subjected to a Pearson correlation analysis against the proportion of dead bees and weight. All statistical analyses were performed using R, version 3.4.3© [69] and IBM SPSS Statistic 25 [70] with the significance level set at p < 0.05 (α of 0.05).

## Results

### Effect of field realistic exposure to clothianidin and/or *V*. *destructor* parasitism on bee survivorship and weight

**Fig 1:**
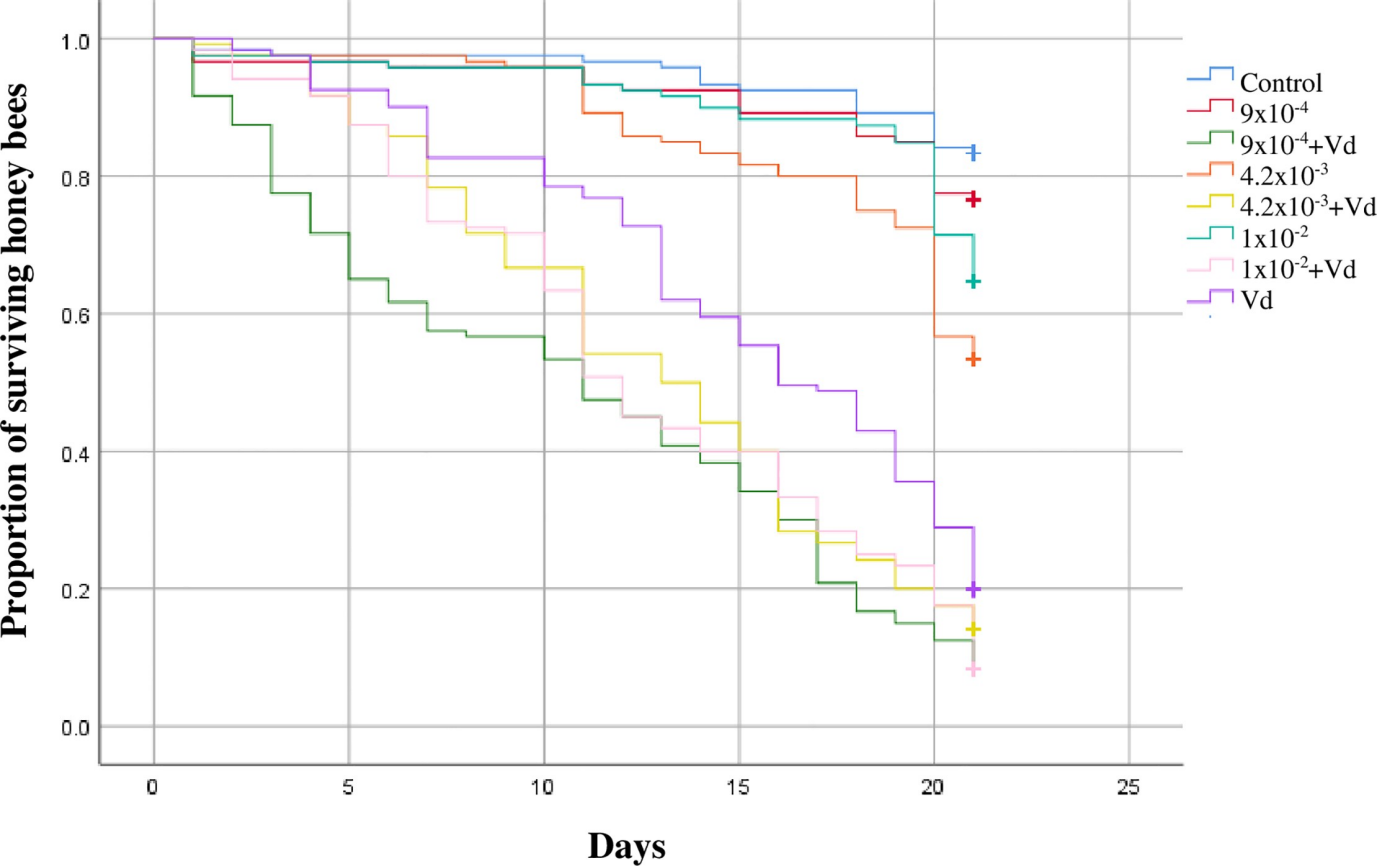
Kaplan-Meier survival curves of adult bees exposed to field realistic doses of clothianidin and/or *V*. *destructor* (Vd) for 21 consecutive days. Log rank (Mantel-Cox) tests were used to determine significant differences between survival curves (adjusted p value of 0.001).

Significant differences in the survival curves were found between treatments (Chi^2^_(7,960)_ = 463, p<0.0001; Fig 1). Pairwise comparisons showed a significant decrease in the proportion of bees that survived between parasitized bees by *V*. *destructor* (0.2) and the control (0.83) (Chi^2^_(1, 240)_ = 103, p<0.0001, V = 0.91). Also, a significant decrease in survivorship was noted in bees exposed to the three doses of clothianidin and parasitized by *V*. *destructor* (0.8, 0.14 and 0.8, respectively) compared to the corresponding dose of clothianidin without *V*. *destructor* (0.77, 0.53 and 0.65, respectively) (Chi^2^_(1, 240)_ = 145 p<0.0001, V = 0.77; Chi^2^_(1, 240)_ = 65, p<0.0001, V = 0.52; Chi^2^_(1, 240)_ = 112, p<0.0001, V = 0.70, respectively). Although a decrease in survivorship occurred in bees exposed to the combined stressors, the main factor affecting survivorship was *V*. *destructor*.

**Fig 2:**
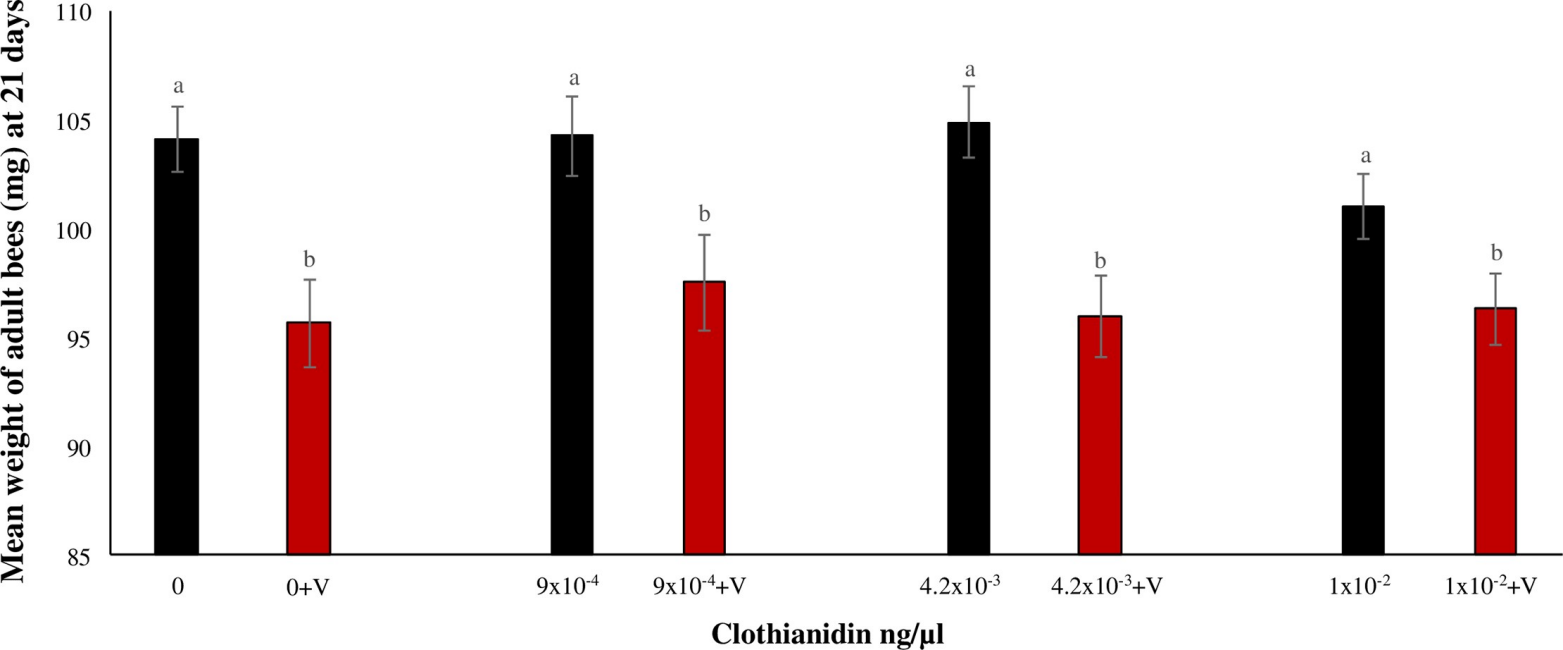
Weight of 21-day old bees after exposure to clothianidin and/or *V*. *destructor*. Mean weight (mg ±S.E.) of adult bees exposed to field realistic doses of clothianidin and/or *V*. *destructor* (V) for 21 consecutive days. Bars with different letters above them represent significant differences using the Tukey’s HSD test after a two-way ANOVA showed significant differences. Non-transformed data are presented.

Adult bee weight was not affected by field realistic doses of clothianidin (F_(3,16)_ = 0.38 p = 0.76, ŋ^2^ = 0.034), but *V*. *destructor* significantly reduced bee weight (F_(1,16)_ = 33.35, p<0.0001, ŋ^2^ = 0.91) (Fig 2). There were no significant differences between treatments of *V*. *destructor* alone and *V*. *destructor* with any clothianidin dose tested, and no interaction between clothianidin dose and *V*. *destructor* on bee weight (F_(3,16)_ = 0.71, p = 0.548, ŋ). Thus, *V*. *destructor* was also the main factor associated with decreased adult weight.

### DWV quantification

**Fig 3:**
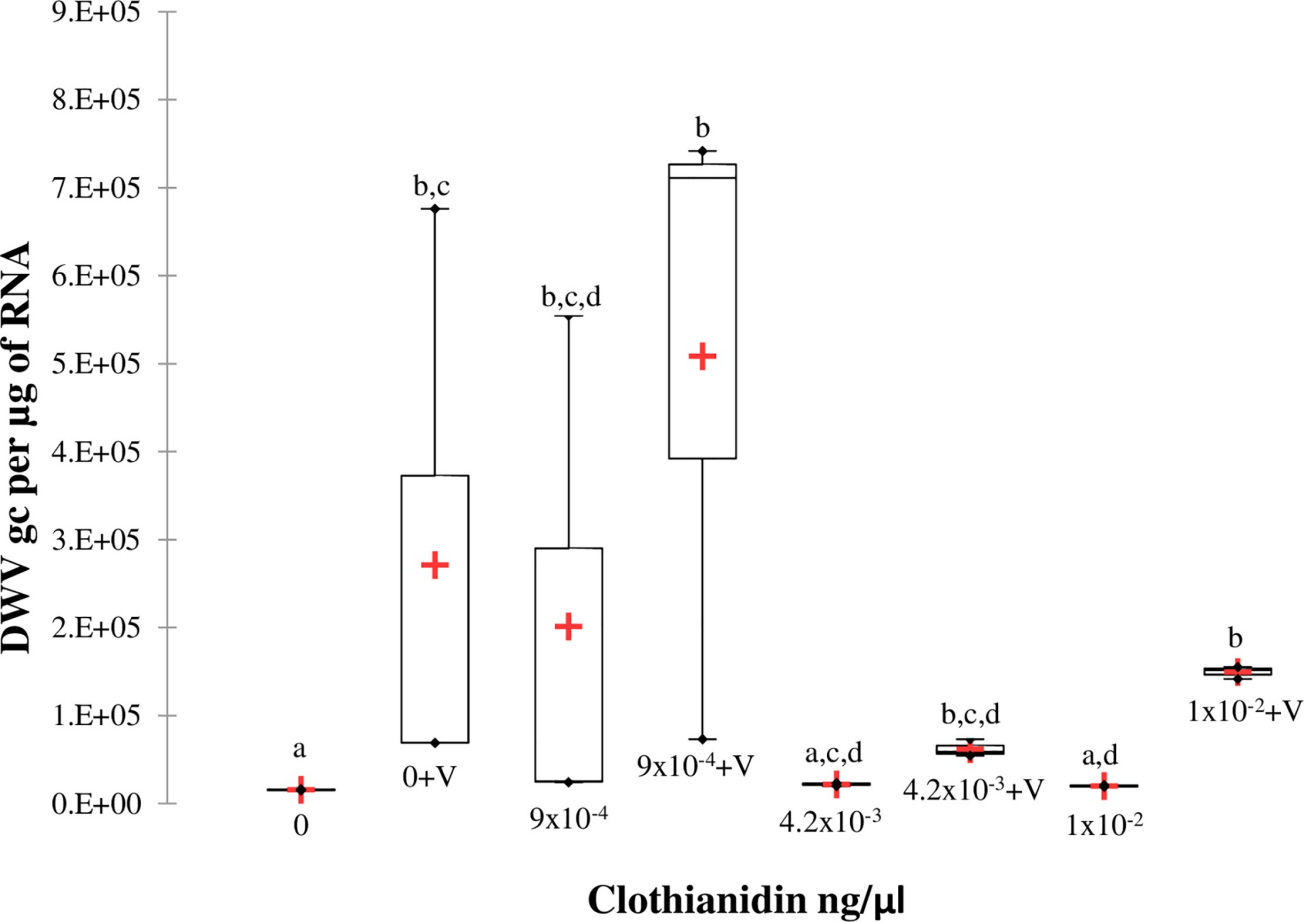
Box plots of DWV levels of 21-day old bees exposed to clothianidin and/or *V*. *destructor*. DWV gc per μg of RNA. Different letters above the boxes represent significant differences using the Conover-Iman procedure after a Kruskal Wallis test showed a significant effect.

A significant effect of clothianidin and *V*. *destructor* on DWV quantification was detected (Chi^2^ = 19.57, p = 0.007, df = 7, ŋ^2^ = 0.78). Among the three field realistic doses of clothianidin alone, only the lowest dose (9x10^-4^ ng/μl) of clothianidin resulted in significantly higher levels of DWV compared to the non-treated control (p<0.05) (Fig 3). However, *V*. *destructor* alone significantly increased DWV levels compared to the non-treated control (p<0.05), but there were no significant differences between the *V*. *destructor* alone treatment and *V*. *destructor* plus clothianidin treatments (p>0.05). Also, there was no interaction between the two factors. Thus, *V*. *destructor* was the main stressor associated with increased DWV levels.

### Gene expression analysis from Quantitative real time-PCR

**Fig 4:**
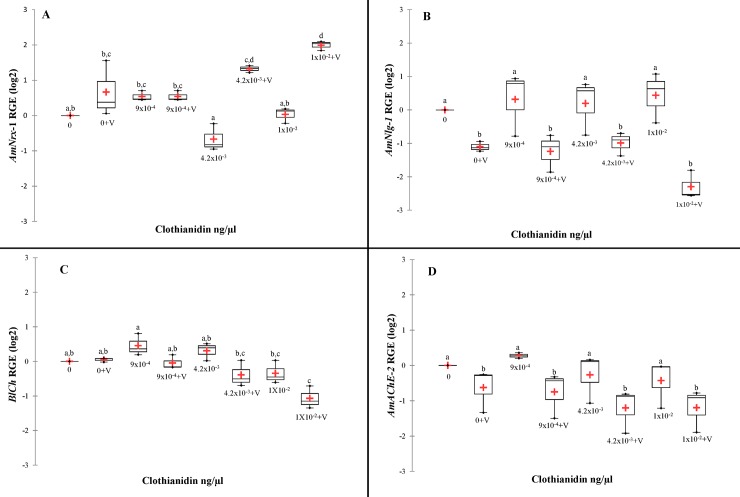
Box plots of the of relative gene expression versus ng of clothianidin. **(A)** *AmNlg-1* relative gene expression, calculated using the Livak 2^ΔΔ^ method with *AmRPS5* as reference gene and 0 ng as calibrator. **(B)** *AmNlg-1* relative gene expression, calculated using the Livak 2^ΔΔ^ method with *AmRPS5* as reference gene and 0 ng as calibrator. **(C)** *BlCh* relative gene expression, calculated using the Livak 2^ΔΔ^ method with *AmRPS5* as reference gene and 0 ng as calibrator. **(D)** *AChE-2* relative gene expression, calculated using the Livak 2^ΔΔ^ method with *AmRPS5* as reference gene and 0 ng as calibrator.

For *AmNrx-1* expression, there was a significant effect of clothianidin (F_(3,16)_ = 5.60, p = 0.008, ŋ^2^ = 0.13; Fig 4A) and *V*. *destructor* (F_(3,16)_ = 72.127, p<0.0001, ŋ^2^ = 0.56), and a significant interaction between the two stressors (F_(3,16)_ = 13.182, p<0.0001, ŋ^2^ = 0.30). The only significant differences in *AmNrx-1* expression from the non-treated control was the up-regulation with 4.2x10^-3^ ng/μl of clothianidin plus *V*. *destructor* and 1x10^-2^ ng/μl of clothianidin plus *V*. *destructor* (p<0.05).

For *AmNlg-1* expression, there was no significant effect by clothianidin (F_(3,16)_ = 0.971, p = 0.431, ŋ^2^ = 0.05; Fig 4B), nor an interaction between clothianidin and *V*. *destructor* (F_(1,16)_ = 2.44, p = 0.102, ŋ^2^ = 0.13), but *AmNlg-1* expression was significantly down-regulated by *V*. *destructor* (F_(1,16)_ = 46.43, p<0.0001, ŋ^2^ = 0.82).

*BlCh* expression was significantly down-regulated by the highest dose of clothianidin (F_(3,16)_ = 13.32, p<0.0001. ŋ^2^ = 0.59; Fig 4C) and by *V*. *destructor* (F_(1,16)_ = 18.50, p = 0.001, ŋ^2^ = 0.28), but no interaction between them was detected (F_(3,16)_ = 2.71, p = 0.080, ŋ^2^ = 0.12).

*AmAChE-2* expression was not affected by clothianidin exposure (F_(3,16)_ = 1.62, p = 0.223, ŋ^2^ = 0.71; Fig 4D), nor by the interaction between clothianidin and *V*. *destructor* (F_(3,16)_ = 0.15, p = 0.926,ŋ^2^ = 0.11), but there was a significant down-regulatory effect by *V*. *destructor* (F_(1,16)_ = 13.51, p = 0.002, ŋ^2^ = 0.17).

### Correlation analyses

Survivorship correlated positively with the expression of *AmNlg-1* (r = 0.745, p<0.0.0001, n = 24), *AmAChE-2* (r = 0.594, p = 0.002, n = 24) and *BlCh* (r = 0.467, p = 0.021, n = 24), and negatively with the expression of *AmNrx-1* (r = -0.589, p = 0.002, n = 24). Weight of adult bees was positively correlated with *BlCh* expression (r = 0.529, p = 0.008, n = 24). No other correlations between survivorship and gene expression or weight and gene expression were significant.

### RNA sequencing

The number of reads per sample were 17,487,602 for the non-treated control, 20,197,182 for 1x10^-2^ ng/μl clothianidin alone, 18,225,872 for *V*. *destructor* alone, and 17,821,269 for 1x10^-2^ ng/μl clothianidin plus *V*. *destructor*. A pairwise read comparison between the non-treated control and 1x10^-2^ ng/μl clothianidin alone identified 264 significantly up-regulated and 306 significantly down-regulated DEGs (p<0.05) (Tables B and C in S1 File), but fewer DEGs significantly up and down-regulated (60 versus 120, respectively) were identified between the non-treated control and *V*. *destructor* alone (p<0.05) (Tables D and E in S1 File). Even fewer significantly differentially regulated DEGs (13 up-regulated and 60 down-regulated) were found from the pairwise comparison between the non-treated control and 1x10^-2^ ng/μl of clothianidin plus *V*. *destructor* (p<0.05) (Tables F and G in S1 File).

In addition to differences in the number of DEGs, treatments also differed in their impact on the expression of the DEGs. For significantly up-regulated DEGs, the average fold change with clothianidin alone was 0.83, *V*. *destructor* alone was 1.78 and the combined stressors was 2.66, indicating that the combined stressors had the greatest degree of impact on those DEGs (Tables B, D and F in S1 File). Similarly, for significantly down-regulated DEGs, the average log fold changes with clothianidin alone was 0.83, *V*. *destructor* alone was 1.86, and the combined stressors was 2.99, indicating that the combined stressors had the highest average degree of impact on those DEGs (Tables C, E and G in S1 File).

### Up and down-regulated DEG pairwise comparisons

**Fig 5:**
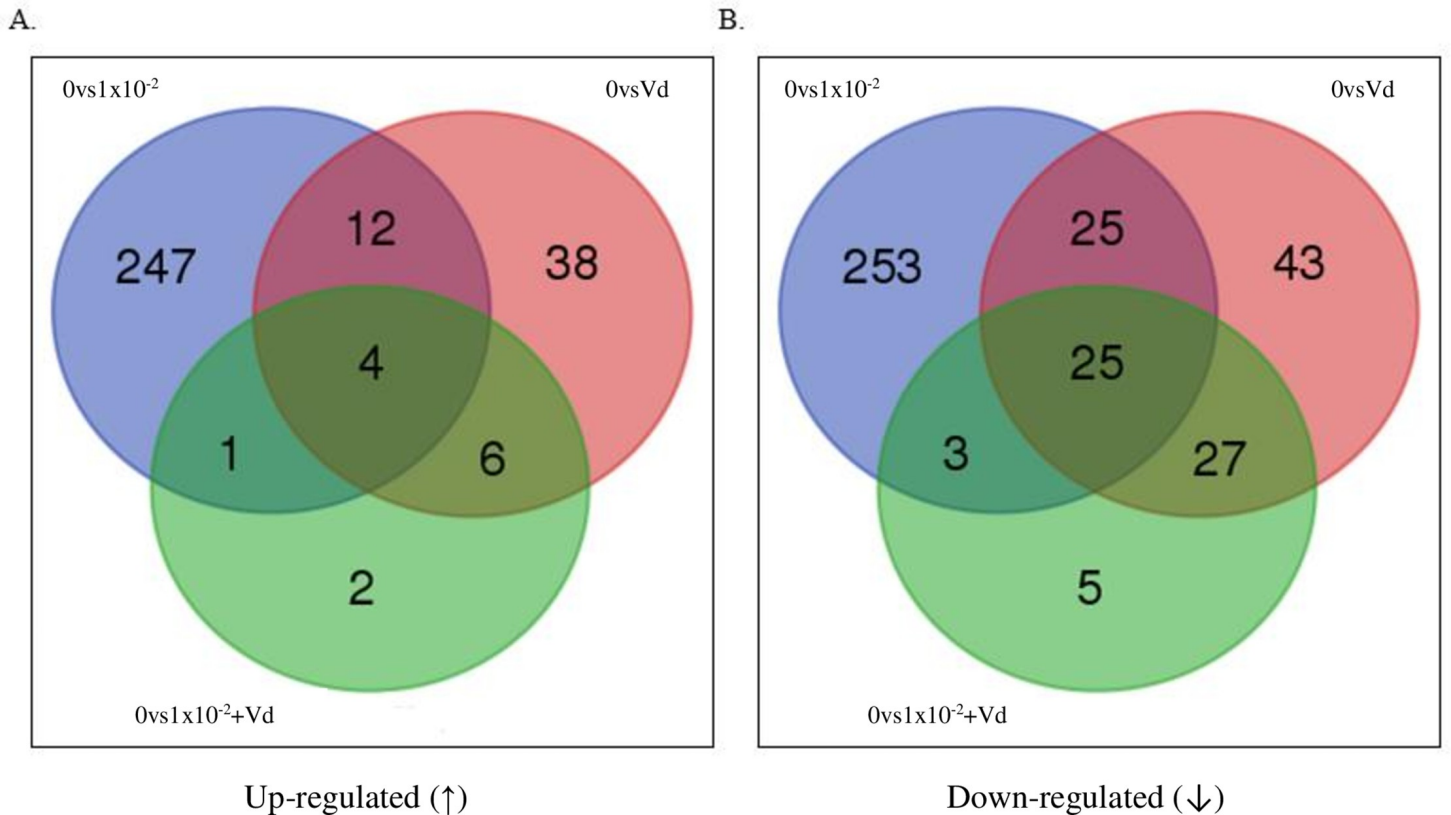
Venn diagram. Number of DEGs in the Differential Expression Analysis (DEA), and the genes in common between the pairwise comparisons of 0 ng/μl of clothianidin vs 1x10^-2^ ng/μl of clothianidin (0vs1x10^-2^), 0 ng/μl vs 0 ng plus *V*. *destructor* (0vsVd) and 0 ng/μl vs 1x10^-2^ ng/μl of clothianidin plus *V*. *destructor* (0vs1x10^-2^+Vd). **(A)** Venn diagram showing the number of up-regulated (↑) DEGs**. (B)** Venn diagram showing the number of down-regulated (↓) DEGs.

The majority of the significantly up-regulated DEGs were unique to clothianidin alone (247 DEGs) followed by those unique to *V*. *destructor* alone (38 DEGs) (Fig 5A and Table H in S1 File), and the two smallest categories were the DEGs shared only between the combined stressors and clothianidin (1 DEG) and unique to the combined stressors (2 DEGs). There were more DEGs shared between *V*. *destructor* and the combined stressors (10 DEGs), than between clothianidin and the combined stressors (5 DEGs). Based on DEG numbers, the results imply a broad impact of clothianidin alone, but there was much less impact with *V*. *destructor* alone, although a higher proportion of those were shared with clothianidin treatment. In contrast, there was very little impact based on DEG number with the combined stressors with relatively low levels of overlap between those DEGs and other treatments.

The majority of the significantly down-regulated DEGs were unique to clothianidin alone (253 DEGs) followed by the DEGs shared by clothianidin alone and *V*. *destructor* alone (50 DEGs) (Fig 5B and Table H in S1 File), and then those unique to *V*. *destructor* alone (43 DEGs). The smallest category was the down-regulated DEGs that were unique to the combined stressors (5 DEGs), which was much less that those shared between *V*. *destructor* and the combined stressors (52 DEGs) or clothianidin and the combined stressors (28 DEGs). Based on the DEG numbers, it appears that the broadest impact was with clothianidin alone, much less with *V*. *destructor* alone and then even less with the combined stressors. The greatest overlap between treatments was for DEGs with *V*. *destructor* and either clothianidin or combined stressors.

### KEGG analysis

Although KEGG analysis assigned less than 20% of all the up or down-regulated DEGs in this study to biological pathways (Tables B-G in S1 File), there were a number of noteworthy KEGG terms among the significantly up or down-regulated DEGs (Tables B-G in S1 File). Some of the KEGG terms unique to DEGs up-regulated by clothianidin alone were Toll like receptor signalling pathway and necroptosis (Table B in S1 File), whereas some KEGG terms unique to DEGs down-regulated by clothianidin alone were glutamatergic synapse and serotonergic synapse (Table C in S1 File). There were KEGG terms for up-regulated DEGs unique with *V*. *destructor* alone for cardiac muscle contraction and arrythmogenic right ventricular cardiomyopathy (Table D in S1 File). Glycolisis/gluconeogenesis and antifolate resistance were KEGG terms associated with down-regulated DEGs unique to *V*. *destructor* alone (Table E in S1 File). While no up-regulated DEGs unique to the combined stressors had KEGG terms (Table F in S1 File), unique down-regulated DEGs by the combined stressors had a KEGG term related to arginine, aspartate and glutamate metabolism (Table G in S1 File). KEGG terms shared between *V*. *destructor* and clothianidin included longevity regulating pathway for worms and apoptosis (Tables B-E in S1 File).

## Discussion

Survivorship was strongly affected by *V*. *destructor* parasitism, whereas field realistic doses of clothianidin alone had a limited effect on it. However, a decrease in the proportion of surviving bees was also significant when bees were exposed to clothianidin plus *V*. *destructor*. Abbo et al. [71] also reported an effect of a neonicotinoid insecticide on honey bee mortality after nine days of oral exposure to a field realistic dose of imidacloprid, which was lower than the 4.2x10^-3^ ng/μl dose of clothianidin used in this study (72 versus 28 times lower than the oral LD_50_, respectively). Many other studies have also shown that *V*. *destructor* parasitism significantly increases individual adult bee mortality [10, 72, 73]. This could be due to *V*. *destructor* piercing the cuticle and membranes of adult bees, secreting saliva and suppressing cellular immunity by preventing wound healing by haemocytes [13, 74, 75]. In addition, the mite can suppress bee humoral immunity, such as by down-regulating antimicrobial peptides (AMPs), like defensin and hymenoptaecin, as well as transmitting viruses to the bee [10, 11, 75, 76, 77]. The effect of clothianidin plus *V*. *destructor* on survivorship indicates that either negative effects on pathways by *V*. *destructor* and clothianidin are being increased by each other, or additional pathways are being affected with the combination. An example of the latter is the KEGG pathway of alanine, aspartate and glutamate metabolism was associated with DEGs down-regulated by the combined stressors but not with either stressor alone. Amino acids, including alanine, aspartate and glutamate, has been linked to immune responses by activating immune cells, regulating cellular redox state and gene expression [78], thus an effect on the metabolism of amino acids could be impacting the ability of the bee to defend herself against pathogens and consequently increasing mortality.

There was no evidence for an interaction between clothianidin and *V*. *destructor* based on weight loss in bees treated during the adult stage, nor was there an effect by clothianidin alone, but there was an effect by *V*. *destructor* alone. Abbo et al. [71] found that honey bees treated with imidacloprid had decreased weight over nine days of exposure. In contrast, weight loss occurs due to *V*. *destructor*, which was proposed to be due to the loss of haemolymph tissue as a result of the mite’s feeding [73]. However, this could also have been due to consumption of fat body during *V*. *destructor* feeding [8]. The fat body is made up of adipocytes, which are cells that store triglycerides (esters from glycerol and three fatty acids), glycogen and protein granules [79]. Thus, an effect due to *V*. *destructor* is not surprising, although the lack of an effect of clothianidin could be due to either no effect on weight or else prolonged exposure allows the bees to compensate for weight loss. The lack of an interaction between *V*. *destructor* and clothianidin exposure is not too surprising if *V*. *destructor* affects weight but clothianidin has no impact upon it, and it appears that feeding by *V*. *destructor* does not alter the lack of an effect of clothianidin on weight.

There was no evidence of an interaction between clothianidin and *V*. *destructor* on levels of DWV in adult bees, but there was an effect of higher DWV levels with the lowest dose of clothianidin alone and with *V*. *destructor* alone. In previous studies on effects of sublethal doses of neonicotinoids to honey bees, clothianidin exposure favoured DWV replication, which was proposed to be due to immunosuppression [56], but thiamethoxam exposure did not affect DWV replication [44]. In this study, only the lowest dose of clothianidin appeared to favor DWV replication, and this suggests that differences between previous studies are a result of a highly dose dependent effect of neonicotinoids on DWV replication. The increase in DWV replication by *V*. *destructor* could be related to the immunosuppression of bees as well as introduction of the virus to the bee, and higher levels of DWV in adult bees parasitized by *V*. *destructor* has been reported many times [10, 11, 75]. The lack of an interaction between clothianidin and *V*. *destructor* on DWV levels indicates that while each stressor has an effect, the combined stressors could have different impacts on the immune responses that do not interact. Moreover, there could be an antagonistic interaction between *V*. *destructor* and clothianidin, as each stressor alone affected DEGs linked to the KEGG pathway for immune responses, like *Vibrio cholerae*, leukocyte transendothelial migration and phagosome, but when combined no DEGs were associated with those biological pathways. These results partially agree with Gregorc et al. [80], who reported an increase in the expression of immune related genes in larvae parasitized by *V*. *destructor* but found no interaction between exposure to imidacloprid and *V*. *destructor*.

Changes in expression of *AmNrx-1* showed an interaction of clothianidin and *V*. *destructor*, as well as an effect due to each stressor alone. *AmNrx-1* encodes for neurexins that are transmembrane proteins located at the presynaptic membrane that help connect neurons during synapse [81]. Neurexins interact with neuroligins by forming a complex during synapse [82]. Up-regulation of *Nrx-1* occurred in bees deprived of sensory stimuli for a prolonged period of time, indicating interference in the formation of the neurexin/neuroligin complex formation during synapse, negatively affecting behaviours and learning [42]. Exposure to the sublethal doses of the neonicotinoid insecticide imidacloprid down-regulated *Nrx-1* expression in brains of *Bombus terrestris*, but effects on behaviours or cognitive processes were not reported [83]. The interaction between clothianidin and *V*. *destructor* indicates that neural connections are more disrupted when the effects of parasitism are combined with field realistic neonicotinoid exposure. The progressive increase in expression with higher concentrations of clothianidin combined with *V*. *destructor* indicates neural connections are being negatively impacted analogous to the damage caused by sensory deprivation, suggesting greater misfunctioning of neural connections. While an up-regulation of *AmNrx-1* as a response to *V*. *destructor* in bees performing self-grooming behaviour has been reported [18], conclusions on the effect of combined stressors on *AmNrx-1* expression associated with self-grooming behaviour cannot be drawn from the results presented in this study. Further investigation of the effects of the combined stressors in *AmNrx-1* expression in bees performing self-grooming behaviour would be valuable to better understand their effect on neural processes related to defence mechanisms.

Expression of *AmNlg-1* showed no interaction of clothianidin and *V*. *destructor*, no effect due to clothianidin, but an effect due to *V*. *destructor* alone. In contrast to *AmNrx-1* expression, *AmNlg-1* expression was down-regulated in sensory deprived bees, possibly due to the reduced stimulation resulting in less *AmNlg-1* expression needed for the regulation of afferent neuronal circuits and neuromuscular motor control, whereas the higher *AmNrx-1* expression may reflect greater apoptosis of neurons in sensory deprived bees [42]. In this study, the greatest effect on expression for both genes was with the highest dose of clothianidin plus *V*. *destructor* with *AmNrx-1* up-regulation and *AmNlg-1* down-regulation. Thus, the relationship between *AmNrx-1* and *AmNlg-1* expression in this study matches that of sensory deprivation. Even though one might expect the stressors to have a stimulatory effect on these genes, the chronic exposure to the stressors over several weeks could explain impacts similar to sensory deprivation. Perhaps the lack of interaction was due to less of an effect of clothianidin on *AmNlg-1* expression than *AmNrx-1* expression, and thus testing higher doses of clothianidin combined with *V*. *destructor* will be able to show that.

*BlCh* expression was not affected by the interaction between clothianidin and *V*. *destructor*, but there was an effect by clothianidin alone and *V*. *destructor* alone. *BlCh* has been associated with neurodegeneration in *Drosophila melanogaster blch* mutants, which showed insoluble protein aggregates throughout the neuropil of the central nervous system [84]. Finley et al. [84] proposed that the protein aggregates throughout the neuropil of *D*. *melanogaster* were comparable to those in human neurodegenerative diseases, such as Alzheimer’s, which has also been linked to the accumulation of insoluble protein aggregates in the central nervous system [85]. The significant down-regulation of *BlCh* expression in bees exposed to the highest dose of clothianidin and *V*. *destructor* could reflect an accumulation of protein aggregates in the neuropil, which could lead to a reduction in brain size due to neural apoptosis, likely impairing neural processes. To the best of our knowledge, this is the first report of the effect of a neonicotinoid on *BlCh* expression. For *V*. *destructor*, only the effects on brood have been examined where Hamiduzzaman et al. [86] found that parasitism did not affect *BlCh* expression. In this study, the expression in adults with *V*. *destructor* alone was almost identical to that of the non-treated control. The progressive decrease in expression with clothianidin and *V*. *destructor* could show that there are increasing levels of aggregates in the brain negatively affecting the central nervous system perhaps like in *D*. *melanogaster blch* mutants. It would be interesting to examine the morphology of the brain of the bee for insoluble protein aggregates with those treatments.

*AmAChE-2* expression did not show an effect from the interaction between clothianidin and *V*. *destructor*, or clothianidin alone, but there was an effect by *V*. *destructor*. *AmAChE-2* catalyzes the breakdown of the neurotransmitter acetylcholine to terminate synaptic transition, and down-regulation or inhibition of acetylcholine esterase results in overstimulation of the central nervous system of insects [87]. Up-regulation of acetylcholine esterase as a response of the binding of clothianidin to nAChRs has been reported by Boily et al. [88] and Alburaki et al. [38]. However, no changes in *AmAChE-2* expression was observed in this study, even though the doses of clothianidin used in the cage experiments by Boily et al. [88] were within the range used in this study (0.03 to 0.24 ng per bee). However, one difference was that the bees in their study were exposed for 10 days, whereas in this study exposure to clothianidin was twice that amount of time. Although there are no reports of the effect of *V*. *destructor* on *AmAChE-2* expression, neurological damage could occur in parasitized bees due to the loss of fat body, which provides energy for the nervous system, and the increase of DWV, which can interfere with an unknown aspect of learning [89]. Because clothianidin had no impact on *AChE-2* expression, the lack of interaction between the two stressors was not unexpected.

Analysis of DEGs was able to show that each stressor and the combined stressors differed in the number of DEGs and the KEGG pathways linked to those DEGs. The highest number of DEGs with up and down-regulation was with clothianidin, followed by *V*. *destructor* and the lowest number with the combined stressors. Rather than an additive or synergistic effect, the combined stressors appear to have an antagonistic effect dramatically reducing the number of up and down-regulated DEGs. This is similar, for example, to the interaction of estrogen receptor modulator, bazedoxifene, and conjugated estrogens that had antagonistic effects on the number of DEGs and KEGG biological pathways in humans [90]. The decreased number of DEGs by the combined stressors could be linked to an impairment of mechanisms used by the bees to compensate for the effect of the stressor alone. Also, unexpected was that there was relatively few DEGs shared by each stressor alone and the combined stressors with the exception of down-regulated DEGs shared between *V*. *destructor* and the combined stressors. One would have expected that many of the DEGs affected by clothianidin or *V*. *destructor* would still be among the DEGs when those stressors were combined with each other. It appears that the effects of *V*. *destructor* were sufficiently widespread to negate most of the up and down-regulatory effects of clothianidin alone and vice versa.

The higher number of up and down-regulated DEGs by clothianidin compared to *V*. *destructor* alone could be an indicator of clothianidin affecting a broader range of metabolic processes. This could be due to neonicotinoids directly affecting the central nervous system [91], which coordinates most of the functions in the various organs of honey bees [92]. In contrast, *V*. *destructor* may have a localized effect, at least during the 21 days of the experiment, at the wound site and the fat body ingested by the parasite. Another possibility is that the bee is more able to compensate for the effects of *V*. *destructor*, thus limiting its impact.

While the number of DEGs differed with the stressors alone and combined, an examination of their associated KEGG pathways was done to determine possible differences in biological pathways. Like the number of DEGs, there were more KEGG pathways affected by clothianidin alone than *V*. *destructor* alone or the combined stressors. Although clothianidin and *V*. *destructor* affected a wide variety of biological pathways, clothianidin seemed to affect more pathways related to energy metabolism, such as insulin secretion and glucagon signaling pathways, also to pathways related to neural disorders, such as morphine addiction, whereas *V*. *destructor* alone affected more pathways linked to cardiac muscle function, like hypertrophic cardiomyopathy. Moreover, *V*. *destructor* and clothianidin shared a number of immune pathways linked to immune responses, like platelet activation and bacterial invasion of epithelial cells, but based on the total number of KEGG pathways affected by the stressors alone, *V*. *destructor* affected a higher percentage of immune pathways compared to clothianidin, and clothianidin affected a higher percentage of pathways related to neural disorders and drug resistance. This agrees with previous studies showing thiamethoxam affecting energy and drug metabolism [37], while *V*. *destructor* affected immune pathways [17]. Unexpectedly, this study found that the combined stressors had no unique DEGs associated immune related pathways or neural disorders, but only one pathway associated with the metabolism of amino acids, indicating an antagonistic effect of the combined stressors, and limited range of novel effects when clothianidin and *V*. *destructor* are combined. The results of the number of DEGs and their associated KEGG pathways with the combined stressors compared to each stressor alone demonstrates how difficult it can be to predict the interaction between stressors.

## Conclusions

**Fig 6:**
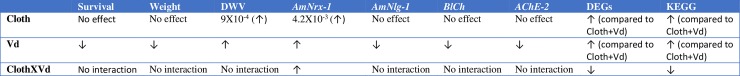
Summary of the effects of clothianidin and/or *V*. *destructor*. Effect of clothianidin (Cloth) and/or *V*. *destructor* (Vd) on honey bee survivorship, weight, DWV levels, the expression of *AmNrx-1*, *AmNlg-1*, *BlCh* and *AmAChE-2*, number of DEGs and KEGG pathways (↑ indicates an increase or up-regulation, ↓ indicates a decrease or a down-regulation).

Clothianidin alone at field realistic doses caused a slight increase in DWV levels and affected a large number of up and down-regulated DEGs in honey bees. *V*. *destructor* parasitism alone, significantly decreased survivorship, increased DWV levels, caused a weight loss and down-regulated three neural related genes (*AmNlg-1*, *BlCh*, and *AmAChE-2*) in the bees, as summarized in Fig 6. One of the main discoveries of this study is the negative effect of *V*. *destructor* on the expression of neural related genes, which is important as *V*. *destructor* parasitism could potentially affect behaviours that are essential for honey bee survival. The number of up and down-regulated DEGs affected by *V*. *destructor* was almost seven times lower than those affected by clothianidin. Interactions between clothianidin and *V*. *destructor* were also noted for survivorship, *AmNrx-1* expression and a reduction in the number of DEGs. The reduced numbers of DEGs and associated KEGG pathways for the combined stressors compared to the stressors alone indicates that the interaction of the stressors is not additive or synergistic, but antagonistic. While previous studies have found a synergistic interaction between neonicotinoid exposure combined with *V*. *destructor* parasitism in overwintering colony mortality [44] or no interaction between neonicotinoid exposure and *V*. *destructor* parasitism in the expression levels of immune related genes [80], this study showed an antagonistic interaction based on the number of DEGs. Overall, the results of this study showed that honey bee health and neural processes could be compromised by exposure to clothianidin or *V*. *destructor*. The combined effect of the stressors is difficult to predict as the reduced numbers of DEGs with the combined stressors could indicate a reduced impact on honey bee health affecting fewer biological pathways associated with the DEGs than with the individual stressors. However, the reduction in the number of DEGs could also indicate a greater effect on biological processes indicating a reduced ability of the bees to compensate for the negative effects of the stressors alone when they are combined, which would result in a negative impact on the colony’s wellness and survival.

## Supporting information

S1 File **Table A. Sequences of PCR primers.** Description of genes, abbreviation, Gene ID, accession number, forward and reverse primers, length of the amplicons (bp), and reference of the target and reference genes used in this study. **Table B. KEGG pathway analysis (0vs1x10-2) of up-regulated DEGs**. KEGG pathways analysis of the DEGs (up-regulated) between the bees treated with 0 ng/μl and 1x10-2 ng/μl of clothianidin (0vs1x10-2). **Table C. KEGG pathway analysis (0vs1x10-2) of down-regulated DEGs**. KEGG pathways analysis of the DEGs (down-regulated) between the bees treated with 0 ng/μl and 1x10-2 ng/μl of clothianidin (0vs1x10-2). **Table D. KEGG pathway analysis (0vsVd) of up-regulated DEGs**. KEGG pathways analysis of the DEGs (up-regulated) between the bees parasitized with *V. destructo*r compared to bees exposed to 0 ng/μl of clothianidin + *V. destructor* (0vsVd). **Table E. KEGG pathway analysis (0vsVd) of down-regulated DEGs**. KEGG pathways analysis of the DEGs (down-regulated) between the bees parasitized with *V. destructo*r compared to bees exposed to 0 ng/μl of clothianidin + *V. destructor* (0vsVd). **Table F. KEGG pathway analysis (0vs1x10-2+Vd) of up-regulated DEGs**. KEGG pathways analysis of the DEGs (up-regulated) between the bees exposed to 1x10-2 ng/μl of clothianidin plus *V. destructor* compared to bees exposed to 0 ng of clothianidin (0vs1x10-2+Vd). **Table G. KEGG pathway analysis (0vs1x10-2+Vd) of down-regulated DEGs**. KEGG pathways analysis of the DEGs (down-regulated) between the bees exposed to 1x10-2 ng/μl of clothianidin plus *V. destructor* compared to bees exposed to 0 ng of clothianidin (0vs1x10-2 ng/μl+Vd). **Table H. Gene IDs in common between pairwise comparisons**. Gene IDs s in common between the pairwise comparisons of 0 ng of clothianidin vs 1x10-2 ng/μl of clothianidin (0vs1x10-2), 0 ng of clothianidin vs *V. destructor* (0vsVd) and 0 ng of clothianidin vs 1x10-2 ng/μl of clothianidin plus *V. destructor* (0vs1x10-2 +Vd).
(PDF)
