# Supplementary material for: Interaction of field realistic doses of clothianidin and Varroa destructor parasitism on adult honey bee (Apis mellifera L.) health and neural gene expression, and antagonistic effects on differentially expressed genes
Source: PLoS One. 2020 Feb 20;15(2):e0229030. doi: 10.1371/journal.pone.0229030 (PMC7032720; doi:10.1371/journal.pone.0229030)
Supplement: S1 File — Table A. Sequences of PCR primers. Description of genes, abbreviation, Gene ID, accession number, forward and reverse primers, length of the amplicons (bp), and reference of the target and reference genes used in this study. Table B. KEGG pathway analysis (0vs1x10-2) of up-regulated DEGs. KEGG pathways analysis of the DEGs (up-regulated) between the bees treated with 0 ng/μl and 1x10^-2^ ng/μl of clothianidin (0vs1x10-2). Table C. KEGG pathway analysis (0vs1x10-2) of down-regulated DEGs. KEGG pathways analysis of the DEGs (down-regulated) between the bees treated with 0 ng/μl and 1x10^-2^ ng/μl of clothianidin (0vs1x10-2). Table D. KEGG pathway analysis (0vsVd) of up-regulated DEGs. KEGG pathways analysis of the DEGs (up-regulated) between the bees parasitized with V. destructor compared to bees exposed to 0 ng/μl of clothianidin + V. destructor (0vsVd). Table E. KEGG pathway analysis (0vsVd) of down-regulated DEGs. KEGG pathways analysis of the DEGs (down-regulated) between the bees parasitized with V. destructor compared to bees exposed to 0 ng/μl of clothianidin + V. destructor (0vsVd). Table F. KEGG pathway analysis (0vs1x10-2+Vd) of up-regulated DEGs. KEGG pathways analysis of the DEGs (up-regulated) between the bees exposed to 1x10^-2^ ng/μl of clothianidin plus V. destructor compared to bees exposed to 0 ng of clothianidin (0vs1x10-2+Vd). Table G. KEGG pathway analysis (0vs1x10-2+Vd) of down-regulated DEGs. KEGG pathways analysis of the DEGs (down-regulated) between the bees exposed to 1x10^-2^ ng/μl of clothianidin plus V. destructor compared to bees exposed to 0 ng of clothianidin (0vs1x10-2 ng/μl+Vd). Table H. Gene IDs in common between pairwise comparisons. Gene IDs s in common between the pairwise comparisons of 0 ng of clothianidin vs 1x10^-2^ ng/μl of clothianidin (0vs1x10-2), 0 ng of clothianidin vs V. destructor (0vsVd) and 0 ng of clothianidin vs 1x10^-2^ ng/μl of clothianidin plus V. destructor (0vs1x10-2 +Vd). (PDF) [file pone.0229030.s001.pdf]

S1 File:

Table A. Sequences of PCR primers. Description of genes, abbreviation, Gene ID, accession number, forward and reverse primers, length of the amplicons (bp), and reference of the target and reference genes used in this study.

Gene description[a]	Abbreviation	Accession number[b]	Primer Forward[c]	Primer Reverse[c]	Amplicon length (bp)	Reference
40S ribosomal protein S5	*AmRPS5*	XM_006570237.2	AATTATTTGGTCGCTGGAATTG	TACCACATTCTGCTGGACGTT	115	Evans [1]
Glyceraldehyde-3-phosphate dehydrogenase 2	*AmGAPD2*	XM_393605.6	GATGCACCCATGTTTGTTTG	TTTGCAGAAGGTGCATCAAC	203	Thomson et al. [2]
Beta actin	*β-actin*	NM_001185146	GATTTGTATGCCAACACTGTCCTT	TTGCATTCTATCTGCGATTCCA	69	Di Prisco et al. [3]
Acetylcholinesterase	*AmAChE-2*	KU532289	GGACATAATGGCGGCTACGA	CTCCTCGCTGTTCGTGAAGT	106	This study
Neurexin 1	*AmNrx-1*	NM-001145740	CTGCTTCGAGCGACGACTAT	ACGACCGGATGGATGATTGG	212	Morfin et al. [4]
Neuroligin 1	*AmNlg-1*	XM_006561837	CCCAATCGTTGGAGGAAGAA	GCATAGCGATTACGGAAGAACTC	69	Biswas et al. [5]
Deformed Wing Virus helicase	*DWV helicase*	AJ489744.2	GCGCTTAGTGGAGGAAATGAA	GCACCTACGCGATGTAAATCTG	69	Di Prisco et al. [3]

[a]Gene description based on the National Center for Biotechnology Information (Bethesda (MD): National Library of Medicine (US), National Center for Biotechnology Information; [1988] – [cited 2017 Apr 06]. Available from: https://www.ncbi.nlm.nih.gov/
[b]Accession number, National Center for Biotechnology Information (Bethesda (MD): National Library of Medicine (US), National Center for Biotechnology Information; [1988] – [cited 2017 Apr 06]. Available from: https://www.ncbi.nlm.nih.gov/
[c]Primers are described from 5’ to 3’

Table B. **KEGG pathway analysis (0vs1x10^{-2}) of up-regulated DEGs**. KEGG pathways analysis of the DEGs (up-regulated) between the bees treated with 0 ng/μl and 1x10^{-2} ng/μl of clothianidin (0vs1x10^{-2}).

**Gene ID**[a]	**Gene description**[b]	**Biological pathway**[c]
GB50276	dual specificity mitogen-activated protein kinase kinase 4	MAPK signaling pathway (ko04010); ErbB signaling pathway (ko04012); TNF signaling pathway (ko04668); Toll-like receptor signaling pathway 9ko04620); Fc epsilon RI signaling pathway (ko04664); GnRH signaling pathway (ko04912); fluid shear stress and atherosclerosis (ko05418); epithelial cell signaling in *Helichobacter pylori* infection (ko05120); HTLV-I infection (ko05166); influenza A (ko05164); hepatitis B (ko05161); Epstein-Barr virus infection (ko05169); Chagas disease (ko05142)
GB42653	glycogenin-1	metabolic pathway (ko01100); starch and sucrose metabolism (ko00500)
GB52079	rapamycin-insensitive companion of mTOR	mTOR signaling pathway (ko04150)
GB51941	fibroblast growth factor 1-like	ras signaling pathway (ko04014); rap1 signaling pathway (ko04015); MAPK signaling pathway (ko04010); hippo signaling pathway-fly (ko04390); PI3K-Akt signaling pathway (ko04151); regulation of actin cytoskeleton (ko04810); pathways in cancer (ko05200); melanoma (ko05218); breast cancer (ko05224)
GB42501	transcription initiation factor TFIID subunit 4-like	basal transcription factors (ko03022); Huntington's disease (ko05016); herpes simplex infection (ko05168)
GB52661	diacylglycerol kinase eta	metabolic pathway (ko01100); biosynthesis of secondary metabolites (ko01110); glycerolipid metabolism (ko00561); glycerophospholipid metabolism (ko0564); phosphatidylinositol signaling system (ko04070); phospholipase D signaling pathway (ko04072); choline metabolism in cancer (ko05231)
GB47802	signal recognition particle receptor subunit beta	protein export (ko03060)
GB41867	endoplasmin	protein processing in endoplasmic reticulum (ko04141); PI3K-Akt signaling pathway (ko04151); IL-17 signaling pathway (ko04657); estrogen signaling pathway (ko04915); thyroid hormone synthesis (ko04918); plant-pathogen interaction (ko04626); pathways in cancer (ko05200); fluid shear stress and atherosclerosis (ko05418)
GB52453	apoptotic protease-activating factor 1-like	apoptosis (ko04210); apoptosis-fly (ko04214); apoptosis-multiple species (ko04215); p53 signaling pathway (ko04115); small cell lung cancer (ko05222); Alzheimer's disease (ko05010); Parkinson's disease (ko05012); amyotrophic lateral sclerosis (ko05014); Huntington's disease (ko05016); legionellosis (ko05134); tuberculosis (ko05152); hepatitis B (ko05161); platinum drug resistance (ko01524)
GB54777	voucher Apme conserved ATPase domain	metabolic pathway (ko01100); pyrimidine metabolism (ko00240); alanine, aspartate and glutamate metabolism (ko00250)
GB43968	uncharacterized	metabolic pathway (ko01100); purine metabolism (ko00230); pyrimidine metabolism (ko00240); RNA polymerase (ko03020)
GB44641	F-box/WD repeat-containing protein 9	vitamin digestion and absorption (ko04977)

GB47605	M-phase phosphoprotein 6	RNA degradation (ko03018)
GB46792	inosine-5'-monophosphate dehydrogenase 1b	metabolic pathway (ko01100); biosynthesis of secondary metabolites (ko01110); propanoate metabolism (ko00640); purine metabolism (ko00230); drug metabolism-other enzymes (ko00983)
GB49571	leucine-rich repeat protein soc-2 homolog	ras signaling pathway (ko04014)
GB54609	condensin complex subunit 1	cell cycle-yeast (ko04111)
GB42920	non-canonical poly(A) RNA polymerase PAPD5-like	RNA degradation (ko03018)
GB52345	cell division cycle 37 homolog	PI3K-Akt signaling pathway (ko04151)
GB44936	histone-arginine methyltransferase	endocrine resistance (ko01522)
GB41989	midasin	ribosome biogenesis in eukaryotes (ko03008)
GB47495	nucleotide exchange factor SIL1	protein processing in endoplasmic reticulum (ko04141)
GB44707	CAAX prenyl protease 2	biosynthesis of antibiotics (ko01130); terpenoid backbone biosynthesis (ko00900)
GB46041	UDP-N-acetylglucosamine--dolichyl-phosphate N-acetylglucosaminephosphotransferase	metabolic pathway (ko01100); N-glycan biosynthesis (ko00510)
GB54242	ribonucleases P/MRP protein subunit POP1	RNA transport (ko03013); ribosome biogenesis in eukaryotes (ko03008)
GB42469	phospholipase B1	metabolic pathway (ko01100); biosynthesis of secondary metabolites (ko01110); ether lipid metabolism (ko00565); arachidonic acid metabolism (ko00590); linoleic acid metabolism (ko00591); alpha-linoleic acid metabolism (ko00592); vitamin digestion and absorption (ko04977)
GB44918	transcription initiation factor TFIID subunit 5	basal transcription factors (ko03022); herpes simplex infection (ko05168)
GB51125	inositol-3-phosphate synthase 1-B	metabolic pathway (ko01100); biosynthesis of antibiotics (ko01130); inositol phosphate metabolism (ko00562); streptomycin biosynthesis (ko00521)
GB47408	histone H2B	viral carcinogenesis (ko05203); systemic lupus erythematosus (ko05322); alcoholism (ko05034)
GB53008	dachsous	hippo signaling pathway-fly (ko04391); hippo signaling pathway-multiple species (ko04392)
GB54372	60 kDa heat shock protein	RNA degradation (ko03018); longevity regulation pathway-worm (ko04212); type I diabetes mellitus (ko04940); legionellosis (ko05134); tuberculosis (ko05152)
GB55144	dynein heavy chain 6	Huntington's disease (ko05016)
GB45046	cell division control protein 6 homolog	cell cycle (ko04110); cell cycle-yeast (ko04111); meiosis-yeast (ko04113)
GB50730	heat shock protein 70Cb ortholog	protein processing in endoplasmic reticulum (ko04141)
GB45913	protein lethal(2)essential for life-like	protein processing in endoplasmic reticulum (ko04141)
GB49117	heat shock 70 kDa protein cognate 3	protein export (ko03060); protein processing in endoplasmic reticulum (ko04141); thyroid hormone synthesis (ko04918); prion disease (ko05020)

GB49250	heme oxygenase	metabolic pathway (ko01100); biosynthesis of secondary metabolites (ko01110); porphyrin and chlorophyll metabolism (ko00860); mineral absorption (ko04978)
GB51481	dual oxidase	MAPK signaling pathway-fly (ko04013); Toll and Imd singling pathway (ko04624)
GB42458	PFF0380w	RNA transport (ko03013)
GB45909	protein lethal(2)essential for life-like	protein processing in endoplasmic reticulum (ko04141)
GB40746	peptidyl-prolyl cis-trans isomerase FKBP4	estrogen signaling pathway (ko04915)
GB48135	L-lactate dehydrogenase-like	metabolic pathway (ko01100); biosynthesis of secondary metabolites (ko01110); microbial metabolism in diverse environment (ko01120); biosynthesis of antibiotics (ko01130); glycolysis/gluconeogenesis (ko00010); pyruvate metabolism (ko00620); cysteine and methionine metabolism (ko00270); glucagon signaling pathway (ko04922)
GB50609	heat shock protein Hsp70Ab-like	spliceosome (ko03040); protein processing in endoplasmic reticulum (ko04141); MAPK signaling pathway (ko04010); endocytosis (ko04144); antigen processing and presentation (ko04612); estrogen signaling pathway (ko04915); legionellosis 9ko05134); measles (ko05162); influenza A (ko05164); Epstein-Barr virus infection (ko05169); toxoplasmosis (ko05145)
GB45910	lethal(2) essential for life-like	protein processing in endoplasmic reticulum (ko04141)
GB48503	polypeptide N-acetylgalactosaminyltransferase 5	metabolic pathway (ko01100); mucin type O-glycan biosynthesis (ko00512)
GB40976	heat shock protein 90	protein processing in endoplasmic reticulum (ko04141); necroptosis (ko04217); PI3K-Akt signaling pathway (ko04151); NOD-like receptor signaling pathway (ko04621); antigen processing and presentation (ko04612); Th17 cell differentiation (ko04659); IL-17 signaling pathway (ko04657); estrogen signaling pathway (ko04915); progesterone-mediated oocyte maturation (ko04914); plant-pathogen interaction (ko04626); pathways in cancer (ko05200); prostate cancer (ko05215); fluid shear stress and atherosclerosis (ko05418)
GB45495	heat shock protein 83	protein processing in endoplasmic reticulum (ko04141); necroptosis (ko04217); PI3K-Akt signaling pathway (ko04151); NOD-like receptor signaling pathway (ko04621); antigen processing and presentation (ko04612); Th17 cell differentiation (ko04659); IL-17 signaling pathway (ko04657); estrogen signaling pathway (ko04915); progesterone-mediated oocyte maturation (ko04914); plant-pathogen interaction (ko04626); pathways in cancer (ko05200); prostate cancer (ko05215); fluid shear stress and atherosclerosis (ko05418)

[a]Gene ID, BeeBase gene identifiers of the Honey bee genome assembly 4.5; http://hymenopteragenome.org

[b]Gene description based on the National Center for Biotechnology Information (Bethesda (MD): National Library of Medicine (US), National Center for Biotechnology Information; [1988] – [cited 2017 Apr 06]. Available from: https://www.ncbi.nlm.nih.gov/

[c]Bioloical pathways and (KO) identifiers from a biological pathway based on KASS search. Available from http://www.genome.jp/kaas-bin/kaas_main [6]

Table C. KEGG pathway analysis (0vs1x10^{-2}) of down-regulated DEGs. KEGG pathways analysis of the DEGs (down-regulated) between the bees treated with 0 ng/µl and 1x10^{-2} ng/µl of clothianidin (0vs1x10^{-2}).

**Gene ID**[a]	**Gene description**[b]	**Biological pathway**[c]
GB54292	carbohydrate sulfotransferase 11-like	glycosaminoglycan biosynthesis-chondoitin sulfate/dermatan sulfate (ko00532)
GB41912	oxidoreductase YrbE-like	metabolic pathway (ko01100); microbial in diverse environments (ko01120); biosynthesis of antibiotics (ko01130); inositol phosphate metabolism (ko00562); streptomycin biosynthesis (ko00521)
GB45213	acyl-CoA synthetase short-chain family member 3	metabolic pathway (ko01100); propanoate metabolism (ko00640)
GB49854	alpha-amylase	metabolic pathway (ko01100); starch and sucrose metabolism (ko00500); carbohydrate digestion and absorption (ko04973)
GB42964	beta-1,3-glucosyltransferase	other types of O-glycan biosynthesis (ko00514)
GB51583	kynurenine/alpha-aminoadipate aminotransferase	metabolic pathway (ko01100); biosynthesis of antibiotics (ko01130); 2-oxocarboxylic acid metabolism (ko01210); biosynthesis of amino acids (ko01230); lysine biosynthesis (ko00300); lysine degradation (ko00310); tryptophan metabolism (ko00380)
GB55499	alkaline phosphatase 4-like	metabolic pathway (ko01100); thiamine metabolism (ko00730); folate biosynthesis (ko00790); two-component system (ko02020)
GB42551	alpha-N-acetylglucosaminidase	metabolic pathway (ko01100); glycosaminoglycan degradation (ko00531); lysosome (ko04142)
GB52359	high affinity cAMP-specific and IBMX-insensitive 3',5'-cyclic phosphodiesterase 8A	purine metabolism (ko00939); morphine addiction (ko05032)
GB55701	aldehyde dehydrogenase family 7 member A1 homolog	metabolic pathway (ko01100); biosynthesis of secondary metabolites (ko01110); microbial in diverse environments (ko01120); biosynthesis of antibiotics (ko01130); biosynthesis of amino acids (ko01230); ascorbate and aldarate metabolism (ko00053); pyruvate metabolism (ko00620); fatty acid degradation (ko00071); glycerolipid metabolism (ko00561); glycine, serine and threonine metabolism (ko00260); valine, leucine and isoleucine degradation (ko00280); lysine biosynthesis (ko00300); lysine degradation (ko00310); arginine and proline metabolism (ko00300); histidine metabolism (ko00340); tryptophan metabolism (ko00380); beta-alanine metabolism (ko00410)
GB42218	acyl-CoA Delta(11) desaturase	fatty acid metabolism (ko01212); biosynthesis of unsaturated fatty acids (ko01040); AMPK signaling pathway (ko04152); PPAR signaling pathway (ko03320); longevity regulating pathway-worm (ko04212)
GB50655	cysteine dioxygenase type 1	metabolic pathway (ko01100); cysteine and methionine metabolism (ko00270); taurine and hypotaurine metabolism (ko00430)
GB55263	fatty acyl-CoA reductase CG5065	cutin, suberine and wax biosynthesis (ko0073); peroxisome (ko04146); longevity regulating pathway-worm (ko04212)
GB41545	MD-2-related lipid-recognition	lysosome (ko04142)

	protein-like	
GB44043	juvenile hormone methyltransferase	insect hormone biosynthesis (ko00981)
GB49929	laminin subunit alpha	metabolic pathway (ko01100); PI3K-Akt signaling pathway (ko04151); ECM-receptor interaction (ko04512); focal adhesion (ko04510); pathways in cancer (ko05200); small cell lung cancer (ko05222); human papillomavirus infection (ko05165); amoebiasis (ko05146); toxoplasmosis (ko05145)
GB49147	argininosuccinate synthase	metabolic pathway (ko01100); biosynthesis of secondary metabolites (ko01110); biosynthesis of antibiotics (ko01130); biosynthesis of amino acids (ko01230); aspartate and glutamate metabolism (ko00250); arginine biosynthesis (ko00220); fluid shear stress and atherosclerosis (ko05418)
GB40362	flap endonuclease 1	DNA replication (ko03030); DNA replication (ko03030); base excision repair (ko03410); non-homologous end-joining (ko03450)
GB51494	voucher SC320 phosphoenolpyruvate carboxykinase	metabolic pathway (ko01100); biosynthesis of secondary metabolites (ko01110); microbial in diverse environments (ko01120); biosynthesis of antibiotics (ko01130); citrate cycle (ko00020); pyruvate metabolism (ko00620); foxO signaling pathway (ko04068); PI3K-Akt signaling pathway (ko04151); AMPK signaling pathway (ko04152); insulin signaling pathway (ko04910); glucagon signaling pathway (ko04922); adipocytokine signaling pathway (ko04920); PPAR signaling pathway (ko03320); proximal tubule bicarbonate reclamation (ko04964); insulin resistance (ko04931)
GB50218	ornithine aminotransferase	metabolic pathway (ko01100); biosynthesis of secondary metabolites (ko01110); biosynthesis of antibiotics (ko01130); arginine and proline metabolism (ko00300)
GB53440	mitochondrial enolase superfamily member 1-like	microbial in diverse environments (ko01120); fructose and mannose metabolism (ko00051)
GB40124	LIM domain-containing protein jub	renin secretion (ko04924); Renin-angiotensin system (ko04614); hypertrophic cardiomyopathy (ko05410); Chagas disease (ko05142)
GB43006	glucose dehydrogenas	metabolic pathway (ko01100); glycine, serine and threonine metabolism (ko00260)
GB40431	beta-ureidopropionase	metabolic pathway (ko01100); pyrimidine metabolism (ko00240); beta-alanine metabolism (ko00410); pantothenate and CoA biosynthesis (ko00770); drug metabolism-other enzymes (ko00983)
GB46444	serine--pyruvate aminotransferase	metabolic pathway (ko01100); biosynthesis of secondary metabolites (ko01110); microbial in diverse environments (ko01120); biosynthesis of antibiotics (ko01130); carbon metabolism (ko01200); glyoxylate and dicarbosylate metabolism (ko00630); methane metabolism (ko00680); aspartate and glutamate metabolism (ko00250); glycine, serine and threonine metabolism (ko00260); peroxisome (ko04146)
GB50871	serine/threonine-protein kinase SIK2	glucagon signaling pathway (ko04922)
GB51238	acyl-CoA Delta(11) desaturase-like	fatty acid metabolism (ko01212); AMPK signaling pathway (ko04152); PPAR signaling pathway (ko03320)
GB47970	alpha-aminoadipic semialdehyde synthase	metabolic pathway (ko01100); biosynthesis of secondary metabolites (ko01110); biosynthesis of antibiotics (ko01130); lysine degradation (ko00310)
GB55070	carbonic anhydrase 2-like	nitrogen metabolism (ko00910)

GB42431	adenylate kinase 1	metabolic pathway (ko01100); biosynthesis of secondary metabolites (ko01110); biosynthesis of antibiotics (ko01130); purine metabolism (ko00939); thiamine metabolism (ko00730)
GB49775	crystallin, alpha B	protein processing in endoplasmic reticulum (ko04141); longevity regulating pathway-multiple species (ko04213)
GB55766	cGMP-dependent protein kinase 1	cGMP-PKG signaling pathway (ko04022); platelet activation (ko04611); vascular smooth muscle contraction (ko04270); salivary secretion (ko04970); long-term depression (ko04730); olfactory transduction (ko04740); circadian entrainment (ko04713)
GB40074	hormone receptor-like in 38	aldosterone synthesis and secretions (ko04925)
GB42526	malate dehydrogenase	metabolic pathway (ko01100); biosynthesis of secondary metabolites (ko01110); microbial in diverse environments (ko01120); biosynthesis of antibiotics (ko01130); carbon metabolism (ko01200); citrate cycle (ko00020); pyruvate metabolism (ko00620); glyoxylate and dicarboxylate metabolism (ko00630); carbon fixation in photosynthetic organisms (ko00710); carbon fixation in photosynthetic organisms (ko00710); cysteine and methionine metabolism (ko00270)
GB48079	trypsin alpha-3	neuroactive ligand-receptor interaction (ko04080); pancreatic secretion (ko04972); protein digestion and absorption (ko04974); influenza A (ko05164)
GB45824	phosphoserine phosphatase	metabolic pathway (ko01100); microbial in diverse environments (ko01120); biosynthesis of antibiotics (ko01130); carbon metabolism (ko01200); biosynthesis of amino acids (ko01230); methane metabolism (ko00680); glycine, serine and threonine metabolism (ko00260)
GB42217	acyl-CoA Delta(11) desaturase-like	fatty acid metabolism (ko01212); AMPK signaling pathway (ko04152); PPAR signaling pathway (ko03320)
GB52857	chitinase-3-like protein 1	metabolic pathway (ko01100); amino sugar and nucleotide sugar metabolism (ko00520)
GB43575	trehalase-like	metabolic pathway (ko01100); starch and sucrose metabolism (ko00500)
GB47200	bifunctional methylenetetrahydrofolate dehydrogenase/cyclohydrolase	metabolic pathway (ko01100); one carbon pool by folate (ko00670)
GB45538	fructose-1,6-bisphosphatase 1	metabolic pathway (ko01100); biosynthesis of secondary metabolites (ko01110); microbial in diverse environments (ko01120); biosynthesis of antibiotics (ko01130); carbon metabolism (ko01200); pentose phosphate pathway (ko0030); fructose and mannose metabolism (ko00051); carbon fixation in phtosynthetic organisms (ko00710); carbon fixation in photosynthetic organisms (ko00710); methane metabolism (ko00680); AMPK signaling pathway (ko04152); insulin signaling pathway (ko04910)
GB55705	inositol monophosphatase 2	metabolic pathway (ko01100); inositol phosphate metabolism (ko00562); streptomycin biosynthesis (ko00521); phosphotidylinositol signaling pathway (ko04070)
GB48474	chitinase 3	metabolic pathway (ko01100); amino sugar and nucleotide sugar metabolism (ko00520)
GB51814	glucose dehydrogenase	metabolic pathway (ko01100); glycine, serine and threonine metabolism (ko00260)

GB41367	histone acetyltransferase KAT8	alanine, aspartate and glutamate metabolism (ko00250); peroxisome (ko04146)
GB41306	actin, clone 205-like	rap1 signaling pathway (ko04015); hippo signaling pathway (ko04390); hippo signaling pathway-fly (ko04391); phagosome (ko04145); apoptosis (ko04210); focal adhesion (ko04510); adherens junction (ko04520); tight junction (ko04530); regulation of actin cytoskeleton (ko04810); platelet activation (ko04611); leukocyte transendothelial migration (ko04670); oxytocin signaling pathway (ko4921); thyroid hormone signaling pathway (ko04919); phototransduction (ko04745); proteoglycans in cancer (ko05205); fluid shear stress and atherosclerosis (ko05418); hypertrophic cardiomyopathy (ko05410); arrhythmogenic right ventricular cardiomyopathy (ko05412); dilated cardiomyopathy (ko05414); viral myocarditis (ko05416); *Vibrio cholerae* infection (ko05110); pathogenic *Escherichia coli* infection (ko05130); *Salmonella* infection (ko05132); Shigellosis (ko05131); bacterial invasion of epithelial cells (ko05100); influenza A (ko05164)
GB46302	1-phosphatidylinositol 4,5-bisphosphate phosphodiesterase-like	metabolic pathway (ko01100); inositol phosphate metabolism (ko00562); rap1 signaling pathway (ko04015); Wnt signalling pathway (ko04310); apelin signaling pathway (ko04371); calcium signaling pathway (ko04020); phosphatidylinositol signaling pathway (ko04070); phospholipase D signaling pathway (ko04072); sphingolipid signaling pathway (ko04071); cGMP-PKG signaling pathway (ko04022); gap junction (ko04540); platelet activation (ko04611); NOD-like receptor signaling pathway (ko04621); chemokine signaling pathway (ko04062); insulin secretion (ko04911); glucagon signaling pathway (ko04922); GnRH signaling pathway (ko04912); estrogen signaling pathway (ko04915); oxytocin signaling pathway (ko4921); thyroid hormone synthesis (ko04918); thyroid hormone signaling pathway (ko04919); melanogenesis (ko04916); renin secretion (ko04924); aldosterone synthesis and secretions (ko04925); adrenergic signaling in cardiomyocytes (ko04261); vascular smooth muscle contraction (ko04270); salivary secretion (ko04970); gastric acid secretion (ko04971); pancreatic secretion (ko04972); endocrine and other factor-regulated calcium reabsorption (ko04961); glutamatergic synapse (ko04724); cholinergic synapse (ko04725); dopaminergic synapse (ko04728); serotonergic synapse (ko04726); long-term potential (ko04720); long-term depression (ko04730); retrograde endocannabinoid signaling (ko04723); phototransduction (ko04745); inflammatory mediator regulation of TRP channels (ko04750); circadian entrainment (ko04713); pathways in cancer (ko05200); Alzheimer's disease (ko05010); Huntington's disease (ko05016); AGE-RAGE signaling pathway in diabetic complications (ko04933); amoebiasis (ko05146); Chagas disease (ko05142); African trypanosomiasis (ko05143)
GB49543	alanine--glyoxylate aminotransferase 2-like	metabolic pathway (ko01100); biosynthesis of unsaturated fatty acids (ko00564)
GB55515	inositol oxygenase	ascorbate and aldarate metabolism (ko00053); inositol phosphate metabolism (ko00562)
GB55765	cGMP-dependent protein kinase 1-like	gap junction (ko04540); platelet activation (ko04611); regulation of liposys in adipocytes (ko04923); vascular smooth muscle contraction (ko04270); salivary secretion (ko04970); long-term depression (ko04730); olfactory transduction (ko04740)

GB47304	5-formyltetrahydrofolate cyclo-ligase	metabolic pathway (ko01100); one carbon pool by folate (ko00670)
GB55537	transketolase	metabolic pathway (ko01100); biosynthesis of secondary metabolites (ko01110); microbial in diverse environments (ko01120); biosynthesis of antibiotics (ko01130); carbon metabolism (ko01200); biosynthesis of amino acids (ko01230); pentose phosphate pathway (ko0030); carbon fixation in photosynthetic organisms (ko00710); carbon fixation in photosynthetic organisms (ko00710); biosynthesis of ansamycins (ko01051)
GB42053	epididymal secretory protein E1-like	lysosome (ko04142)
GB45596	elongation of very long chain fatty acids	biosynthesis of secondary metabolites (ko01110); fatty acid metabolism (ko01212); fatty acid elongation (ko0071); biosynthesis of unsaturated fatty acids (ko01040)
GB40261	gamma-interferon-inducible-lysosomal thiol reductase	antigen processing and presentation (ko04612)
GB49845	uncharacterized	amino sugar and nucleotide sugar metabolism (ko00520)
GB45300	interference hedgehog-like	Hedgehog signaling pathway (ko04341)
GB48308	pyruvate dehydrogenase E1 component subunit alpha	metabolic pathway (ko01100); biosynthesis of secondary metabolites (ko01110); microbial in diverse environments (ko01120); biosynthesis of antibiotics (ko01130); carbon metabolism (ko01200); citrate cycle (ko00020); pyruvate metabolism (ko00620); HIF-1 signaling pathway (ko04066); glucagon signaling pathway (ko04922); central carbon metabolic in cancer (ko05230)
GB46579	glucose-6-phosphate 1-dehydrogenase	metabolic pathway (ko01100); biosynthesis of secondary metabolites (ko01110); microbial in diverse environments (ko01120); biosynthesis of antibiotics (ko01130); carbon metabolism (ko01200); pentose phosphate pathway (ko0030); glutathione metabolism (ko00480); central carbon metabolic in cancer (ko05230)
GB47995	BMP and activin membrane-bound inhibitor homolog	Wnt signalling pathway (ko04310); TGF-beta signaling pathway (ko04350)
GB46737	N-acetylgalactosaminyltransferase 6-like	metabolic pathway (ko01100); biosynthesis of secondary metabolites (ko01110); microbial in diverse environments (ko01120); biosynthesis of antibiotics (ko01130); carbon metabolism (ko01200); glyoxylate and dicarboxylate metabolism (ko00630); peroxisome (ko04146)

[a]Gene ID, BeeBase gene identifiers of the Honey bee genome assembly 4.5; http://hymenopteragenome.org ;
[b]Gene description based on the National Center for Biotechnology Information (Bethesda (MD): National Library of Medicine (US), National Center for Biotechnology Information; [1988] – [cited 2017 Apr 06]. Available from: https://www.ncbi.nlm.nih.gov/
[c]Bioloical pathways and (KO) identifiers from a biological pathway based on KASS search. Available from http://www.genome.jp/kaas-bin/kaas_main [6].

Table D. **KEGG pathway analysis (0vsVd) of up-regulated DEGs.** KEGG pathways analysis of the DEGs (up-regulated) between the bees parasitized with *V. destructo*r compared to bees exposed to 0 ng/µl of clothianidin + *V. destructor* (0vsVd).

**Gene ID**[a]	**Gene description**[b]	**Biological pathway**[c]
GB53872	elongation of very long chain fatty acids protein 6-like	biosynthesis of secondary metabolites (ko01110); fatty acid metabolism (ko01212); fatty acid elongation (ko0062); biosynthesis of unsaturated fatty acids (ko01040)
GB47737	glycogen-binding subunit 76A	insulin signalling pathway (ko04910); insulin resistance (ko04931)
GB45910	lethal(2)essential for life-like	protein processing in endoplasmic reticulum (ko04141); longevity regulating pathway-multiple species (ko04213)
GB45763	tropomyosin-2-like	cardiac muscle contraction (ko04260); adrenergic signaling in cardiomyocytes (ko04261); hypertrophic cardiomyopathy (ko05410); dilated cardiomyopathy (ko05414)
GB48999	helix-loop-helix protein 11	proteoglycans in cancer (ko05205)
GB41311	actin, alpha skeletal muscle-like	rap1 signaling pathway (ko04015); hippo signaling pathway (ko04390); hippo signaling pathway-fly (ko04391); phagosome (ko04145); apoptosis (ko04210); focal adhesion (ko04510); adherens junction (ko04520); tight junction (ko04530); regulation of actin cytoskeleton (ko04810); platelet activation (ko04611); leukocyte trans endothelial migration (ko04670); oxytocin signaling pathway (ko04921); thyroid hormone signaling pathway (ko04919); photo transduction-fly (ko04745); proteoglycans in cancer (ko05205); fluid shear stress and atherosclerosis (ko05418); hypertrophic cardiomyopathy (ko05410); arrhythmogenic right ventricular cardiomyopathy (ko05412); dilated cardiomyopathy (ko05414); viral myocarditis (ko05416); *Vibrio cholerae* infection (ko05110); pathogenic *Escherichia coli* infection (ko05130); *Salmonella* infection (ko05132); shigellosis (ko05131); bacterial invasion of epithelial cells (ko05100); influenza A (ko05164)
GB48079	trypsin alpha-3	neuroactive ligand-receptor interaction (ko04080); pancreatic secretion (ko04972); protein digestion and absorption (ko04974); influenza A (ko05164)
GB44139	calmodulin-lysine N-methyltransferase	lysine degradation (ko00310)
GB51095	cryptochrome 2	circadian rhythm (ko04710)

[a]Gene ID, BeeBase gene identifiers of the Honey bee genome assembly 4.5; http://hymenopteragenome.org ;
[b]Gene description based on the National Center for Biotechnology Information (Bethesda (MD): National Library of Medicine (US), National Center for Biotechnology Information; [1988] – [cited 2017 Apr 06]. Available from: https://www.ncbi.nlm.nih.gov/
[c]Bioloical pathways and (KO) identifiers from a biological pathway based on KASS search. Available from http://www.genome.jp/kaas-bin/kaas_main [6].

Table E. KEGG pathway analysis (0vsVd) of down-regulated DEGs. KEGG pathways analysis of the DEGs (down-regulated) between the bees parasitized with *V. destructo*r compared to bees exposed to 0 ng/µl of clothianidin + *V. destructor* (0vsVd).

**Gene ID**[a]	**Gene description**[b]	**Biological pathway**[c]
GB44610	AMP deaminase 2	metabolic pathway (ko01100); biosynthesis of secondary metabolites (ko01110); biosynthesis of antibiotics (ko01130); purine metabolism (ko00230)
GB50655	cysteine dioxygenase type 1	metabolic pathway (ko01100); cysteine and methionine metabolism (ko00270); taurine and hypotaurine metabolism (ko00430)
GB47618	defensin 2	Toll and Imd signaling pathway (ko04624)
GB55701	aldehyde dehydrogenase family 7 member A1 homolog	metabolic pathway (ko01100); biosynthesis of secondary metabolites (ko01110); microbial metabolism in diverse environments (ko01120); biosynthesis of antibiotics (ko01130); glycolysis/gluconeogenesis (ko00010); ascorbate and alderate metabolism (ko00053); pyruvate metabolism (ko00620); fatty acid degradation (ko00071); glycerolipid metabolism (ko00561); glycine, serine and threonine metabolism (ko00260); valine, leucine and isoleucine degradation (ko00280); lysine degradation (ko00310); arginine and proline metabolism (ko00330); histidine metabolism (ko00340); tryptophan metabolism (ko00380); beta-alanine metabolism (ko00410)
GB54678	sodium-coupled neutral amino acid transporter 9 homolog	mTOR signaling pathway (ko04150)
GB44455	uncharacterized	Toll and Imd signaling pathway (ko04624)
GB49147	argininosuccinate synthase	metabolic pathway (ko01100); biosynthesis of secondary metabolites (ko01110); biosynthesis of antibiotics (ko01130); biosynthesis of amino acids (ko01230); alanine, aspartate and glutamate metabolism (ko00250); arginine biosynthesis (ko00220); fluid shear stress and atherosclerosis (ko05418)
GB41545	mellifera MD-2-related lipid-recognition protein-like	lysosome (ko04142)
GB54097	malvolio	lysosome (ko04142); ferroptosis (ko04216); mineral absorption (ko04978)
GB42964	beta-1,3-glucosyltransferase	other types of O-glycan biosynthesis (ko00514)
GB51238	acyl-CoA Delta(11) desaturase-like	fatty acid metabolism (ko01212); biosynthesis of unsaturated fatty acids (ko01040); AMPK signaling pathway (ko04152); PPAR signaling pathway (ko03320); longevity regulating pathway-worm (ko04212)
GB49086	folylpolyglutamate synthase	metabolic pathway (ko01100); folate biosynthesis (ko00790); antifolate resistance (ko01523)
GB55638	tryptophan 2,3-dioxygenase	metabolic pathway (ko01100); tryptophan metabolism (ko00380)

[a]Gene ID, BeeBase gene identifiers of the Honey bee genome assembly 4.5; http://hymenopteragenome.org ;
[b]Gene description based on the National Center for Biotechnology Information (Bethesda (MD): National Library of Medicine (US), National Center for Biotechnology Information; [1988] – [cited 2017 Apr 06]. Available from: https://www.ncbi.nlm.nih.gov/
[c]Bioloical pathways and (KO) identifiers from a biological pathway based on KASS search. Available from http://www.genome.jp/kaas-bin/kaas_main [6].

Table F. **KEGG pathway analysis (0vs1x10^{-2}+Vd) of up-regulated DEGs.** KEGG pathways analysis of the DEGs (up-regulated) between the bees exposed to 1x10^{-2} ng/µl of clothianidin plus *V. destructor* compared to bees exposed to 0 ng of clothianidin (0vs1x10^{-2}+Vd).

**Gene ID**[a]	**Gene description**[b]	**Biological pathway**[c]
GB53872	elongation of very long chain fatty acids protein 6-like	biosynthesis of secondary metabolites (ko01110); fatty acid metabolism (ko01212); fatty acid elongation (ko00062); biosynthesis of unsaturated fatty acids (ko01040)
GB42469	phospholipase B1	metabolic pathway (ko01100); biosynthesis of secondary metabolites (ko01110); glycerophospholipid metabolism (ko00564); ether lipid metabolism (ko00565); arachidonic acid metabolism (ko00590); linoleic acid metabolism (ko00591); alpha-linoleic acid metabolism (ko00592); vitamin digestion and absorption (ko04977)

[a]Gene ID, BeeBase gene identifiers of the Honey bee genome assembly 4.5; http://hymenopteragenome.org ;
[b]Gene description based on the National Center for Biotechnology Information (Bethesda (MD): National Library of Medicine (US), National Center for Biotechnology Information; [1988] – [cited 2017 Apr 06]. Available from: https://www.ncbi.nlm.nih.gov/
[c]Bioloical pathways and (KO) identifiers from a biological pathway based on KASS search. Available from http://www.genome.jp/kaas-bin/kaas_main [6].

Table G. **KEGG pathway analysis (0vs1x10^{-2}+Vd) of down-regulated DEGs**. KEGG pathways analysis of the DEGs (down-regulated) between the bees exposed to 1x10^{-2} ng/µl of clothianidin plus *V. destructor* compared to bees exposed to 0 ng of clothianidin (0vs1x10^{-2} ng/µl+Vd).

**Gene ID**[a]	**Gene description**[b]	**Biological pathway**[c]
GB44610	AMP deaminasa 2	metabolic pathway (ko01100); biosynthesis of antibiotics (ko0113); biosynthesis of secondary metabolites (ko01110); purine metabolism (ko00230)
GB47618	defensin 2	Toll and Imd signaling pathway (ko04624)
GB50655	cysteine dioxygenase type 1	metabolic pathway (ko01100); cysteine and methionine metabolism (ko00270); taurine and hypotaurine metabolism (ko00430)
GB41912	oxidoreductase YrbE-like	metabolic pathway (ko01100); biosynthesis of antibiotics (ko0113); microbial metabolism in diverse environments (ko01120); streptomycin biosynthesis (ko00521); inositol phosphate metabolism (ko00562)
GB43516	phospholipase A1 member A-like	glycerolipid metabolism (ko00561); Alzheimer's disease (ko05010); PPAR signaling pathway (ko03320)
GB55701	aldehyde dehydrogenase family 7 member A1 homolog	metabolic pathway (ko01100); biosynthesis of antibiotics (ko0113); biosynthesis of secondary metabolites (ko01110); biosynthesis of amino acids (ko01230); glycine, serine and threonine metabolism (ko00260); microbial metabolism in diverse environments (ko01120); glycerolipid metabolism (ko00561); valine, leucine and isoleucine degradation (ko00280); lysine degradation (ko00310); beta-alanine metabolism (ko00410); glycolisis/gluconeogenesis (ko00010); arginine and proline metabolism (ko00330); fatty acid degradation (ko00071); ascorbate and alderate metabolism (ko00053); tryptophan metabolism (ko00380); pyruvate metabolism (ko00620); lysine biosynthesis (ko00300); histidine metabolism (ko00340)
GB43006	glucose dehydrogenase	metabolic pathway (ko01100); glycine, serine and threonine metabolism (ko00260)
GB49147	argininosuccinate synthase	metabolic pathway (ko01100); biosynthesis of antibiotics (ko0113); biosynthesis of secondary metabolites (ko01110); biosynthesis of amino acids (ko01230); fluid shear stress and atherosclerosis (ko05418); alanine, aspartate and glutamate metabolism (ko00250); arginine biosynthesis (ko00220)
GB44455	uncharacterized	Toll and Imd signaling pathway (ko04624)
GB51673	dynein beta chain	Huntington's disease (ko05016)
GB54678	sodium-coupled neutral amino acid transporter 9 homolog	mTOR signaling pathway (ko04150)

[a]Gene ID, BeeBase gene identifiers of the Honey bee genome assembly 4.5; http://hymenopteragenome.org ;
[b]Gene description based on the National Center for Biotechnology Information (Bethesda (MD): National Library of Medicine (US), National Center for Biotechnology Information; [1988] – [cited 2017 Apr 06]. Available from: https://www.ncbi.nlm.nih.gov/
[c]Bioloical pathways and (KO) identifiers from a biological pathway based on KASS search. Available from http://www.genome.jp/kaas-bin/kaas_main [6].

Table H. **Gene IDs in common between pairwise comparisons**. Gene IDs s in common between the pairwise comparisons of 0 ng of clothianidin vs $1x10^{-2}$ ng/µl of clothianidin ($0vs1x10^{-2}$), 0 ng of clothianidin vs *V. destructor* (0vsVd) and 0 ng of clothianidin vs $1x10^{-2}$ ng/µl of clothianidin plus *V. destructor* ($0vs1x10^{-2}$ +Vd).

Pairwise comparisons	Up-regulated	Down-regulated
$0vs1X10^{-2}$, 0vsVd, $0vs1X10^{-2}$+Vd	GB45954, GB47040, GB54343, GB55205	GB53732, GB50655, GB47565, GB42410, GB46984, GB47279, GB47521, GB55452, GB41428, GB47805, GB49147, GB47318, GB50423, GB42703, GB50481, GB42802, GB42146, GB47804, GB54945, GB48289, GB52294, GB52528, GB41361, GB55701, GB51306
$0vs1X10^{-2}$, 0vsVd	GB45907, GB51884, GB40266, GB54493, GB52146, GB42492, GB55204, GB45913, GB50238, GB45910, GB55029, GB46001	GB49940, GB42964, GB44070, GB46814, GB52184, GB40164, GB55406, GB53798, GB53354, GB45746, GB51238, GB48576, GB52837, GB40248, GB51146, GB41545, GB42704, GB51383, GB43508, GB48260, GB52642, GB41212, GB44476, GB54315, GB51441
$0vs1X10^{-2}$, $0vs1X10^{-2}$+Vd	GB42469	GB43006 GB41912 GB53888
$0vs1X10^{-2}$+Vd, 0vsVd	GB42475, GB42798, GB53872, GB53641, GB41096, GB46226	GB54506, GB44455, GB40635, GB54678, GB46367, GB47885, GB47546, GB40148, GB51698, GB46368, GB42623, GB49440, GB47771, GB49441, GB43515, GB44344, GB48029, GB51345, GB48746, GB47618, GB47563, GB51223, GB42468, GB42981, GB48662, GB43500, GB44610,
$0vs1X10^{-2}$	GB51885, GB40976, GB48311, GB41925, GB52791, GB41026, GB47678, GB52010, GB55461, GB51606, GB40810, GB45280, GB44513, GB54185, GB51948, GB55470, GB42754, GB47624, GB52453, GB55436, GB48171, GB47595, GB44707, GB45644, GB55072, GB47331, GB45597, GB49117, GB43822, GB47484, GB45861, GB52592, GB41973, GB44611, GB48503, GB50276, GB47603, GB41293, GB43784, GB53073, GB42766, GB52345, GB55989, GB43193, GB55592, GB42891, GB46339, GB50836, GB46458, GB42744, GB44510, GB44782, GB53008, GB41989, GB44416, GB47802, GB49571, GB51481, GB54969, GB46534, GB44056, GB51941, GB40401, GB40972,	GB54292, GB49796, GB41646, GB40124, GB52515, GB48198, GB42433, GB49543, GB50975, GB41306, GB46557, GB51658, GB51494, GB40379, GB50822, GB47637, GB54517, GB55511, GB49394, GB55765, GB47304, GB45300, GB53371, GB45725, GB50026, GB53120, GB43580, GB49929, GB46853, GB44112, GB43518, GB51467, GB42053, GB44988, GB42607, GB41946, GB43727, GB44043, GB47995, GB53925, GB40284, GB46302, GB46444, GB44074, GB40092, GB55864, GB48474, GB55835, GB50761, GB41418, GB43576, GB53014, GB48936, GB52489, GB47200, GB52857, GB52766, GB50061, GB50871, GB51435, GB49775, GB47148, GB51650, GB53115,

	GB48833, GB42897, GB46774, GB55149, GB48086, GB52854, GB42317, GB45872, GB50115, GB51125, GB52245, GB45363, GB40340, GB50816, GB45040, GB50313, GB41443, GB42690, GB41326, GB47469, GB41300, GB44918, GB43504, GB47495, GB50673, GB40967, GB44923, GB53620, GB50520, GB43968, GB41983, GB40746, GB53974, GB53209, GB41042, GB41034, GB51638, GB42466, GB42020, GB54609, GB54777, GB45495, GB50130, GB41867, GB46060, GB52097, GB53558, GB45403, GB51263, GB45909, GB45351, GB41352, GB54665, GB49250, GB42920, GB47408, GB50756, GB53068, GB44936, GB52043, GB41281, GB46762, GB50848, GB49715, GB47648, GB52079, GB52989, GB42424, GB51602, GB45159, GB44677, GB53048, GB54404, GB43962, GB51680, GB44098, GB43783, GB51849, GB42501, GB54441, GB51436, GB41136, GB53200, GB40721, GB47964, GB47538, GB44308, GB53043, GB42888, GB50441, GB42959, GB48860, GB50033, GB53443, GB44804, GB54974, GB45764, GB54242, GB54752, GB48823, GB41660, GB47292, GB51122, GB48922, GB40507, GB45906, GB41884, GB51984, GB55071, GB48631, GB53221, GB53454, GB53604, GB50865, GB49651, GB54048, GB54133, GB41215, GB50730, GB41290, GB46041, GB41969, GB52794, GB48360, GB46051, GB52661, GB44348, GB42245, GB46563, GB50288, GB46792, GB42653, GB41117, GB55223, GB53793, GB53369, GB54372, GB50442, GB50340, GB54420, GB40719, GB41181, GB46306, GB48135, GB50297, GB42458, GB52583, GB51123, GB44734, GB49069, GB54610, GB45046, GB50857, GB47409, GB46072, GB44641, GB55666, GB52560, GB52490, GB54832, GB53244, GB47605, GB46429, GB51047, GB46261, GB51772, GB50609, GB55202, GB43074, GB55593, GB40519, GB49385,	GB40681, GB54260, GB53965, GB48905, GB40074, GB46858, GB48256, GB54313, GB55499, GB48079, GB53067, GB51834, GB40806, GB51814, GB49966, GB48391, GB55207, GB49888, GB44452, GB47482, GB46737, GB55766, GB49258, GB44552, GB49544, GB46225, GB40639, GB52907, GB52308, GB52359, GB45824, GB47819, GB42769, GB45927, GB51098, GB42427, GB53625, GB41367, GB42526, GB41033, GB47506, GB46366, GB48738, GB46289, GB52581, GB42985, GB48087, GB50977, GB53372, GB46301, GB55445, GB48146, GB53987, GB44457, GB55209, GB50744, GB45596, GB48147, GB52318, GB42962, GB49004, GB50453, GB43823, GB40136, GB49706, GB53769, GB52441, GB40114, GB48917, GB50000, GB42640, GB44967, GB54942, GB46579, GB52446, GB50890, GB48483, GB54390, GB48831, GB51583, GB50862, GB40163, GB54356, GB50005, GB42218, GB45174, GB49361, GB40683, GB40362, GB46294, GB40212, GB53887, GB48308, GB54806, GB55729, GB53024, GB44024, GB48109, GB41719, GB40261, GB55537, GB49845, GB55393, GB55000, GB47943, GB47004, GB40493, GB56028, GB40521, GB52004, GB42431, GB40615, GB43311, GB41331, GB42931, GB46749, GB45681, GB54996, GB46304, GB43342, GB49286, GB45213, GB52186, GB43509, GB43129, GB55203, GB50596, GB55705, GB42261, GB54507, GB49802, GB51979, GB55263, GB51732, GB51840, GB40285, GB53440, GB42351, GB46276, GB53716, GB45855, GB42807, GB54167, GB42217, GB50924, GB54153, GB50272, GB43688, GB50149, GB45464, GB53261, GB50116, GB47970, GB45538, GB51371, GB51567, GB42586, GB51515, GB43871, GB46309, GB49854, GB54486, GB42609, GB40431, GB42262, GB51888, GB41945, GB55070, GB42801, GB50290, GB55515, GB50449,

	GB55144, GB45339, GB44599, GB50141, GB47770, GB45122, GB51736, GB55617, GB55541, GB47934, GB46314	GB41497, GB42343, GB42551, GB44477, GB49848, GB40945, GB54941, GB42252, GB50450, GB40218, GB43575, GB49259, GB53114, GB51733, GB42626, GB50218, GB43447
0vsVd	GB54396, GB51095, GB46557, GB41806, GB44139, GB49887, GB43360, GB50117, GB52910, GB53120, GB50526, GB42652, GB49462, GB52492, GB48079, GB48858, GB41869, GB48999, GB46842, GB41097, GB53503, GB40684, GB44633, GB47737, GB40286, GB47946, GB50047, GB40337, GB55206, GB41311, GB50674, GB51727, GB47215, GB45088, GB45763, GB50290, GB52144, GB49790	GB54804, GB49086, GB51790, GB53876, GB45861, GB53037, GB46620, GB49386, GB54097, GB47545, GB55149, GB50749, GB50550, GB43879, GB51379, GB50509, GB49442, GB55846, GB48577, GB50629, GB45609, GB52162, GB45584, GB54460, GB52542, GB43783, GB48407, GB47520, GB48105, GB43805, GB50880, GB43392, GB48310, GB43231, GB41735, GB46817, GB48663, GB51001, GB54954, GB52361, GB55638, GB48790, GB46428
0vs1X10^{-2}+Vd	GB55208, GB53576	GB51673, GB54238, GB51989, GB43516, GB55613

References

1. Evans JD. Beepath: an ordered quantitative-PCR array for exploring honey bee immunity and disease. J. Invertebr. Pathol.. 2006 Oct 1;93(2):135-9.

2. Thompson GJ, Yockey H, Lim J, Oldroyd BP. Experimental manipulation of ovary activation and gene expression in honey bee (Apis mellifera) queens and workers: testing hypotheses of reproductive regulation J. Exp. Zool. B Mol. Dev. Evol. 2007 Oct 1;307(10):600-10.

3. Di Prisco G, Cavaliere V, Annoscia D, Varricchio P, Caprio E, Nazzi F, et al. Neonicotinoid clothianidin adversely affects insect immunity and promotes replication of a viral pathogen in honey bees. Proc. Natl. Acad. Sci. USA. 2013 Nov 12;110(46):18466-71.

4. Morfin N, Given K, Evans M, Guzman-Novoa E, Hunt GJ. Grooming behavior and gene expression of the Indiana “mite-biter” honey bee stock. Apidologie. 2019 Dec 17:1-9. doi.org/10.1007/s13592-019-00710-y

5. Biswas S, Reinhard J, Oakeshott J, Russell R, Srinivasan MV, Claudianos C. Sensory regulation of neuroligins and neurexin I in the honeybee brain. PloS One. 2010 Feb 9;5(2):e9133.

6. Moriya Y, Itoh M, Okuda S, Yoshizawa AC, Kanehisa M. KAAS: an automatic genome annotation and pathway reconstruction server. Nucleic Acids Res. 2007 Jul 1;35(suppl_2):W182-5.doi: 10.1093/nar/gkm321.
